# Generalized extinction of fear memory depends on co-allocation of synaptic plasticity in dendrites

**DOI:** 10.1038/s41467-023-35805-9

**Published:** 2023-01-31

**Authors:** Zhiwei Xu, Erez Geron, Luis M. Pérez-Cuesta, Yang Bai, Wen-Biao Gan

**Affiliations:** 1 Institute of Neurological and Psychiatric Disorders, Shenzhen Bay Laboratory, Shenzhen, 518132 China; 2 Peking University Shenzhen Graduate School, Shenzhen, 518055 China; 3 Skirball Institute, Department of Neuroscience and Physiology, New York University School of Medicine, New York, NY 10016 USA

**Keywords:** Cellular neuroscience, Learning and memory

## Abstract

Memories can be modified by new experience in a specific or generalized manner. Changes in synaptic connections are crucial for memory storage, but it remains unknown how synaptic changes associated with different memories are distributed within neuronal circuits and how such distributions affect specific or generalized modification by novel experience. Here we show that fear conditioning with two different auditory stimuli (CS) and footshocks (US) induces dendritic spine elimination mainly on different dendritic branches of layer 5 pyramidal neurons in the mouse motor cortex. Subsequent fear extinction causes CS-specific spine formation and extinction of freezing behavior. In contrast, spine elimination induced by fear conditioning with >2 different CS-USs often co-exists on the same dendritic branches. Fear extinction induces CS-nonspecific spine formation and generalized fear extinction. Moreover, activation of somatostatin-expressing interneurons increases the occurrence of spine elimination induced by different CS-USs on the same dendritic branches and facilitates the generalization of fear extinction. These findings suggest that specific or generalized modification of existing memories by new experience depends on whether synaptic changes induced by previous experiences are segregated or co-exist at the level of individual dendritic branches.

## Abstract (web summary)

How memories are modified by new experiences remain elusive. Here, authors show that specific or generalized modification of memories depends on whether synaptic changes associated with previous memories are segregated or co-exist in dendritic branches.

## Introduction

Memories stored in neuronal circuits are often not independent of each other. A variety of studies show that the modification or update of one memory by new experience could affect another memory^1–11^. Previous studies have suggested that when different memories are encoded in overlapping neuronal populations, the modification of one memory tends to be generalized to the other one^10–16^. However, different memories could also be stored within the same neuronal populations with minimum interference^17–21^, suggesting overlapping neuronal populations encoding different memories does not necessarily lead to generalized memory modification.

Many lines of evidence indicate that experience-dependent changes in synaptic strength and number are critical for memory encoding and storage^22–28^. Different experiences can induce synaptic changes in either distinct neuronal populations^29,30^ or different dendritic branches/segments of the same neuronal population^31–33^. It has been suggested that the distribution of synaptic changes within neurons may affect memory storage capacity and memory recall^34–40^. Nevertheless, whether and how the distribution of synaptic changes associated with different memories at the level of neurons or dendrites affects their being modified by new experience in a specific or generalized manner is not known.

In this study, we examined the remodeling of postsynaptic dendritic spines induced by fear conditioning using different auditory tones (CSs) paired with footshocks (USs) and by subsequent fear extinction in the mouse motor cortex, a region involved in fear extinction generalization. We show that fear conditioning with each of different CS-US pairings induces dendritic spine elimination on a subset of dendritic branches in layer 5 pyramidal neurons. When spine elimination induced by different CS-US pairings co-exists on the same dendritic branches, fear extinction induces CS non-specific spine formation and causes generalized reduction of freezing responses. Our findings reveal the importance of segregated or intermingled distribution of synaptic changes at the level of dendritic branches in specific or generalized memory modification.

## Results

### Motor cortex is important for fear extinction generalization

**Fig. 1:**
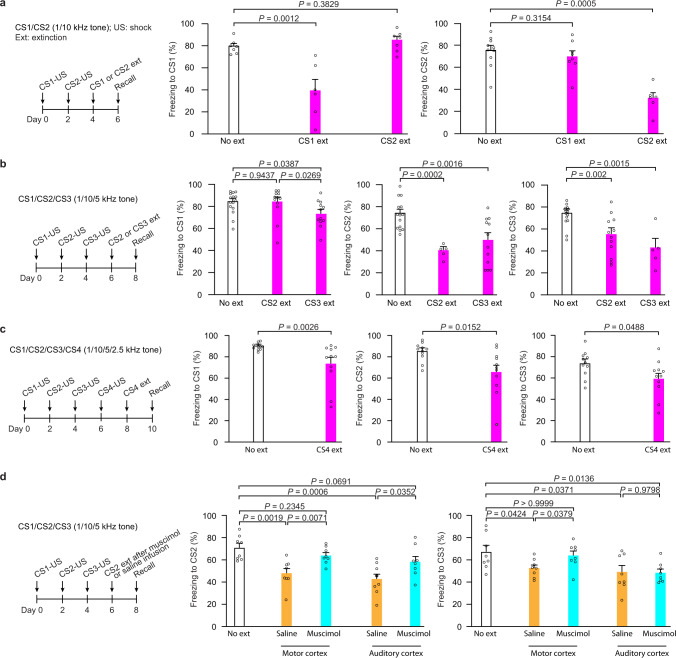
The motor cortex is important for fear extinction generalization. **a** Mice (YFP-H line) were subjected to CS1-US and CS2-US pairings followed by CS1 or CS2 extinction (CS1: 1 kHz; CS2: 10 kHz). The freezing response to CS1 after CS2 extinction was comparable to that after no extinction (*P* = 0.3829, Mann–Whitney *U* test; *n* = 7, 6, and 7 mice in no extinction, CS1 extinction, and CS2 extinction groups respectively). No significant difference in the freezing response to CS2 between CS1 extinction and no extinction groups (*P* = 0.3154, Mann–Whitney *U* test; *n* = 10, 8 and 6 mice in no extinction, CS1 extinction and CS2 extinction groups respectively). *P* values for comparison between groups were calculated using Mann–Whitney *U* test and shown in the graph. **b** Mice (YFP-H line) were subjected to CS1-US, CS2-US, and CS3-US pairings followed by CS2 or CS3 extinction (CS1: 1 kHz; CS2: 10 kHz; CS3: 5 kHz). After CS2 extinction, the freezing response to CS3 was significantly lower than that in no extinction group (*P* = 0.002, unpaired *t* test; *n* = 15, 12 and 5 mice in no extinction, CS2 extinction and CS3 extinction groups respectively). After CS3 extinction, the freezing response to CS1 and CS2 was lower than that in no extinction group (CS1: *P* = 0.0269; *n* = 15, 12, and 12 mice in no extinction, CS2 extinction and CS3 extinction groups respectively; CS2: *P* = 0.0016; *n* = 14, 5, and 12 mice in no extinction, CS2 extinction and CS3 extinction groups respectively; unpaired *t* test). Statistical analyses used were unpaired *t* test for mice receiving CS2 extinction and CS1 or CS3 recall or mice receiving CS3 extinction and CS1 or CS2 recall and Mann–Whitney *U* test for mice receiving CS2 extinction and CS2 recall or mice receiving CS3 extinction and CS3 recall. **c** Mice (YFP-H line) were subjected to CS1-US, CS2-US, CS3-US, and CS4-US pairings followed by CS4 extinction (CS4: 2.5 kHz). After CS4 extinction, the freezing response to CS1, CS2, or CS3 was lower than that in no extinction group (CS1, *P* = 0.0026; CS2 and CS3, *P* < 0.0152 and 0.0488, respectively; unpaired *t* test; *n* = 11 mice in each group). *P* values for comparison between groups were calculated using unpaired *t* test. **d** Mice (YFP-H line) were subjected to three different CS-US pairings followed by CS2 extinction (CS1: 1 kHz; CS2: 10 kHz; CS3: 5 kHz). Muscimol or vehicle was infused bilaterally into the auditory or primary motor cortex before CS2 extinction. The freezing response to CS3 after CS2 extinction in mice with muscimol infusion into the motor cortex was significantly higher than that in vehicle-treated group (*P* < 0.0379, Mann–Whitney U test), and comparable to that in no extinction group (*P* > 0.9999, Mann–Whitney U test; *n* = 8 mice in each group). *P* values for comparison between groups were calculated using Mann–Whitney *U* test. Error bars, ±S.E.M. All statistical tests were performed two-sided.

To investigate how the distribution of experience-dependent synaptic changes might affect their being modified by subsequent new experience, we took advantage of auditory-cued fear conditioning and extinction paradigms in which both specific and generalized extinction of fear memories were observed in humans and animal models^5–9^. When mice were subjected to two different auditory tones (CSs) paired with foot-shocks (USs), fear extinction with one CS did not significantly reduce the freezing response to the other CS, indicating CS-specific fear extinction (Fig. 1a and Supplementary Fig. 1; CS: 1, 2.5, 5, or 10 kHz tone). In contrast, in mice subjected to three different CSs paired with US, fear extinction with CS3 significantly reduced the freezing response to CS1 and CS2 (Fig. 1b; CS1: 1 kHz, CS2: 10 kHz, CS3: 5 kHz; *P* < 0.05 and *P* < 0.01, respectively; Supplementary Fig. 2). Fear extinction with CS2 also reduced the freezing response to CS3 (Fig. 1b; *P* < 0.01). In addition, when mice were subjected to four different CSs paired with US (CS1: 1 kHz, CS2: 10 kHz, CS3: 5 kHz, CS4: 2.5 kHz), fear extinction with CS4 significantly reduced the freezing response to CS1, CS2, and CS3 (Fig. 1c and Supplementary Fig. 3; CS1: *P* < 0.01; CS2 and CS3: *P* < 0.05). Together, these results indicate that mice exhibit CS-specific fear extinction when received two different CS-US pairings, but generalized fear extinction when received 3–4 different CS-US pairings.

Many brain regions including auditory and motor cortices are found to be important for fear conditioning and extinction^41–48^. To investigate whether these cortical regions are involved in fear extinction generalization, we inactivated either the auditory or primary motor cortex with muscimol before CS2 extinction and tested the effect of CS2 extinction in mice subjected to three different CS-US pairings (Fig. 1d; CS1: 1 kHz, CS2: 10 kHz, CS3: 5 kHz). Consistent with previous studies^47,48^, muscimol inactivation of the auditory cortex or the motor cortex before CS2 extinction training prevented the reduction in the freezing response to CS2, but not to CS1, when compared to saline-injected controls (Fig. 1d and Supplementary Fig. 4; *P* < 0.05 for the auditory cortex and *P* < 0.01 for the motor cortex). Notably, muscimol inactivation of the primary motor cortex, not the auditory cortex, prevented the generalized reduction in the freezing response to CS3 after CS2 extinction (Fig. 1d; *P* < 0.05, compared to saline controls). These results suggest that the primary motor cortex is potentially involved in the generalization of fear extinction in mice subjected to three different CS-US pairings.

### Fear conditioning and extinction-induced dendritic spine elimination and formation likely contribute to changes in neuronal activity and fear responses

In line with previous studies^48^, we found that fear conditioning induced dendritic spine elimination whereas fear extinction induced new spine formation on apical dendrites of layer 5 pyramidal neurons in the primary motor cortex (Supplementary Fig. 5a–c, f–h). Furthermore, the degree of spine elimination or formation correlated with the level of freezing after fear conditioning or extinction respectively (Supplementary Fig. 5d, e, i, j).

As the output of a neuron critically depends on the total number of synaptic inputs which excite that neuron^49–51^, the elimination and formation of spines likely affect neuronal activity after fear conditioning and extinction. Consistent with this notion and previous studies^48^, when Ca^2+^ imaging was performed to measure activity changes of layer 5 pyramidal neurons (Supplementary Fig. 6a, b), we found that fear conditioning reduced while fear extinction increased somatic Ca^2+^ activity in response to CS in the motor cortex (Supplementary Fig. 6c, d; total Δ*F*/*F*_0_ during CS exposure: before fear conditioning, 3.69 ± 0.73; after fear conditioning, 1.66 ± 0.52; after fear extinction, 2.92 ± 0.55; *n* = 54, 64 and 64 neurons from 3 to 4 mice, respectively). Notably, the activity level of these layer 5 pyramidal neurons in response to CS were inversely correlated with freezing responses after fear conditioning or extinction (Supplementary Fig. 6e; *P* < 0.05, Pearson’s correlation). Together, these results suggest that fear conditioning or extinction-induced spine elimination or formation may lead to the decreased or increased activity of layer 5 pyramidal neurons, thereby affecting the level of freezing in response to CS.

**Fig. 2:**
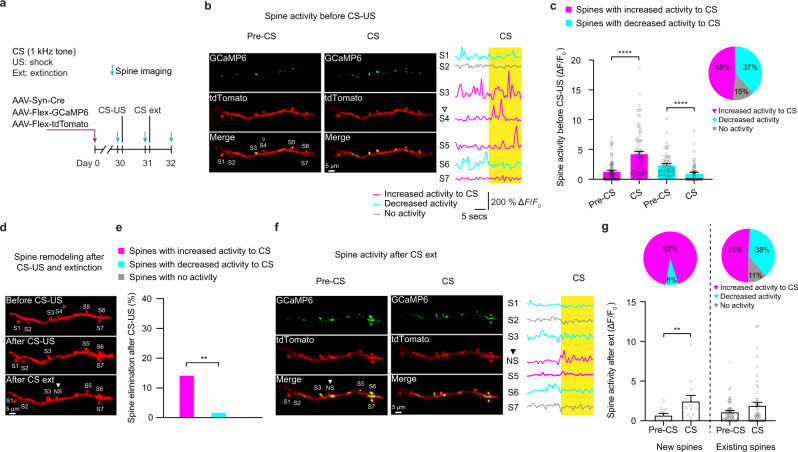
Fear conditioning and extinction-induced dendritic spine elimination and formation of layer 5 pyramidal neurons in the motor cortex likely contribute to neuronal activity changes. **a** Schematic of experimental design. Spine structure and Ca^2+^ activity were examined with or without CS (1 kHz) presentation before and after CS-US pairing and CS extinction. **b** Left and middle panels: Representative images of spine activity on apical dendrites of GCaMP6 and tdTomato co-expressing layer 5 pyramidal neurons with or without CS presentation before fear conditioning. Right panel: GCaMP6 fluorescence traces of seven spines on the left panel without and with CS presentation. Yellow bar denotes the period of CS presentation. Experiments were repeated independently on 178 spines in 6 mice with similar results. **c** Percentage of spines showing increased or decreased activity or no activity in response to CS before fear conditioning. Spines were considered showing increased or decreased responses to CS if total Δ*F*/*F*_0_ of spines during CS/pre-CS presentation period was ≥1 or <1, respectively (during CS vs. pre-CS, *P* < 0.0001 respectively, Wilcoxon matched-pairs signed rank test). Total 178 spines were analyzed. **d** Spine elimination and formation after fear conditioning and extinction. The hollow triangle indicates the spine (S4) eliminated on a dendrite (**b**) after fear conditioning. The solid triangle indicates the newly-formed spine (NS) after fear extinction. Experiments were repeated independently on 178 spines in 6 mice with similar results. **e** After CS-US pairing, spines with increased activity in response to CS had a higher elimination rate than spines with decreased activity to CS (~14.0% vs. 1.5%, *P* < 0.0062, chi-square test; *n* = 85 and 66 spines respectively from 6 mice). **f** Images and Ca^2+^ fluorescence traces of spines on the same dendrite (in **b** and **d**) before and during CS presentation after fear extinction. The solid triangle indicates the newly-formed spine (NS) after fear extinction. Experiments were repeated independently on 13 spines in 6 mice with similar results. **g** After CS extinction, newly-formed spines, but not existing spines, showed a higher activity level during CS presentation when compared to pre-CS presentation period (new spines: *P* = 0.0061; existing spines: *P* = 0.1167; Wilcoxon matched-pairs signed rank test; *n* = 13 and 53 spines respectively). A larger percentage of newly-formed spines than existing spines also showed increased activity in response to CS (~92% vs. 51%, *P* = 0.0066, chi-square test). Error bars, ±S.E.M. All statistical tests were performed two-sided. ** *P* < 0.01; **** *P* < 0.0001.

To gain insights into how fear conditioning and extinction-induced spine elimination and formation affect neuronal activity, we performed Ca^2+^ imaging of dendritic spines of layer 5 pyramidal neurons in the motor cortex of mice subjected to fear conditioning and extinction (Fig. 2a). Before fear conditioning, ~48% and 37% of spines showed increased (total Δ*F*/*F*_0_ during CS/pre-CS ≥ 1) and decreased Ca^2+^ activity in response to the CS presentation respectively (Fig. 2b, c; total Δ*F*/*F*_0_ of spines with increased activity: pre-CS, 1.33 ± 0.19; during CS, 4.27 ± 0.40; total Δ*F*/*F*_0_ of spines with decreased activity: pre-CS, 2.39 ± 0.23; during CS, 0.99 ± 0.19). Notably, after fear conditioning, spines with increased activity had a significantly higher elimination rate over a period of 24 h as compared to spines with decreased activity (Fig. 2d, e; elimination rate: ~14% vs 1.5%, *P* < 0.01, chi-square test; *n* = 85 and 66 spines, respectively from six mice). Furthermore, after fear extinction, newly-formed spines, but not existing spines, showed a higher level of Ca^2+^ activity during the CS presentation than during pre-CS (Fig. 2f, g; total Δ*F*/*F*_0_ of new spines: pre-CS, 0.70 ± 0.23; CS exposure, 2.49 ± 0.71; *P* < 0.01. Total Δ*F*/*F*_0_ of existing spines: pre-CS, 1.12 ± 0.19; CS exposure, 1.94 ± 0.40; *n* = 13 new and 53 existing spines from six mice, respectively). The fraction of newly-formed spines with increased activity in response to CS was significantly larger than that of existing spines (Fig. 2g; ~92% vs 51%, *P* < 0.01, chi-square test). These observations suggest that existing spines with increased activity in response to CS were more likely to be eliminated after fear conditioning, while new spines formed after fear extinction were preferentially active in response to CS, thereby contributing to changes of neuronal activity and freezing behaviors.

### Spine elimination induced by different CS-US pairings shows overlapping distribution at the level of individual neurons

Given the functional impacts of fear conditioning and extinction-induced spine remodeling in the motor cortex and the involvement of this cortical region in fear extinction generalization, we next sought to investigate how spine elimination induced by different CS-US pairings are distributed on layer 5 pyramidal neurons and whether such distribution is related to specific or generalized modification of these cells by fear extinction stimuli.

**Fig. 3:**
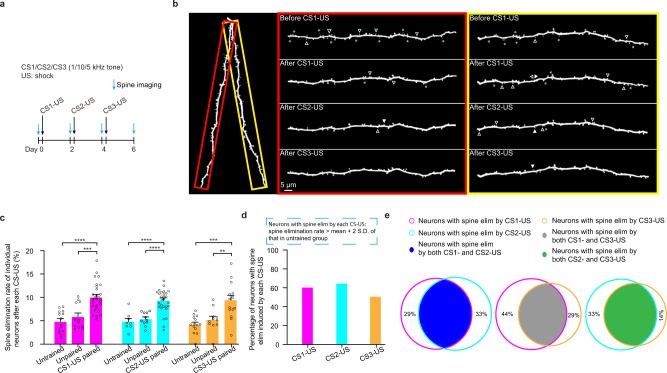
Distribution of spine elimination induced by different CS-US pairings on individual neurons. **a** Experimental design to examine spine elimination induced by different CS-USs in the motor cortex of YFP-H line mice (CS1: 1 kHz; CS2: 10 kHz; CS3: 5 kHz). **b** Representative images of spine elimination induced by three different CS-US pairings on apical dendritic branches from same neuron in YFP-H line mice. The hollow triangles indicate the spines eliminated in the succeeding image. The solid triangles indicate the newly-formed spines when compared to the preceding image. The asterisks indicate filopodia. Experiments were repeated independently on 36 branches in 14 mice with similar results. **c** Comparison of spine elimination rates of individual neurons (circles) between mice subjected to different CS-US pairings and untrained or unpaired control mice (CS1-US pairing vs. untrained or unpaired, *P* < 0.0001 or *P* = 0.0003, respectively; n = 28, 14 and 12 neurons respectively. CS2-US pairing vs. untrained or unpaired, *P* < 0.0001; *n* = 28, 8 and 11 neurons respectively. CS3-US pairing vs. untrained or unpaired, *P* = 0.0001 and *P* = 0.0036 respectively; *n* = 14, 14 and 11 neurons respectively; Mann–Whitney U test). **d** After each CS-US pairing, the majority of Layer 5 pyramidal neurons showed the spine elimination rate higher than the mean plus two times the standard deviation of that in untrained control mice. **e** Percentage of overlap between neurons with spine elimination induced by two different CS-US pairings. The percentage in the non-overlapping area indicates the proportion of non-overlapping neurons relative to neurons with spine elimination induced by each of the different CS-US pairings. Error bars, ±S.E.M. All statistical tests were performed two-sided. ** *P* < 0.01; *** *P* < 0.001; **** *P* < 0.0001.

We first determined whether dendritic spine elimination induced by fear conditioning with two or three different CS-US pairings (CS1: 1 kHz, CS2: 10 kHz, CS3: 5 kHz; each separated by 2 days) was distributed on distinct or overlapping populations of layer 5 pyramidal neurons (Fig. 3a, b). Individual neurons with spine elimination induced by each CS-US pairing were defined as those with the spine elimination rate higher than the mean plus 2 times the standard deviation of that in untrained control mice (average number of spines per neuron: CS1-US pairing, 58 ± 3 from 28 mice; CS2-US pairing, 55 ± 3 from 28 mice; CS3-US pairing, 47 ± 4 from 14 mice). We found that >50% of layer 5 pyramidal neurons showed spine elimination induced by each of three different CS-US pairings (Fig. 3c, d; spine elimination rate: CS1-US pairing, 10.0 ± 0.6%; untrained, 4.8 ± 0.6%; unpaired, 5.9 ± 0.8%; Pairing vs. untrained, *P* < 0.0001; Pairing vs. unpaired, *P* < 0.001; *n* = 28, 14, and 12 neurons, respectively. CS2-US pairing, 9.1 ± 0.5%; untrained, 4.8 ± 0.7%; unpaired, 5.4 ± 0.4%; Pairing vs. untrained or unpaired, *P* < 0.0001; n = 28, 8, and 11 neurons, respectively. CS3-US pairing, 9.2 ± 1.1%; untrained, 4.2 ± 0.5%; unpaired, 5.2 ± 0.7%; Pairing vs. untrained or unpaired, *P* < 0.001 and *P* < 0.01, respectively; *n* = 14, 14, and 11 neurons, respectively). Notably, ~71% of neurons with spine elimination induced by CS1-US pairing also showed spine elimination induced by CS2-US pairing (Fig. 3e; total 28 neurons after CS1-US and CS2-US pairings). ~56% or ~67% of neurons with spine elimination induced by CS1-US pairing or CS2-US pairing also showed spine elimination induced by CS3-US pairing (Fig. 3e; total 14 neurons after CS1-US and CS3-US pairings or CS2-US and CS3-US pairings). These results indicate that spine elimination induced by different CS-US pairings is distributed in a largely overlapping neuronal population, regardless of CS-specific or generalized fear extinction condition.

### Spine elimination induced by 2 and 3 different CS-US pairings shows differential dendritic distribution

**Fig. 4:**
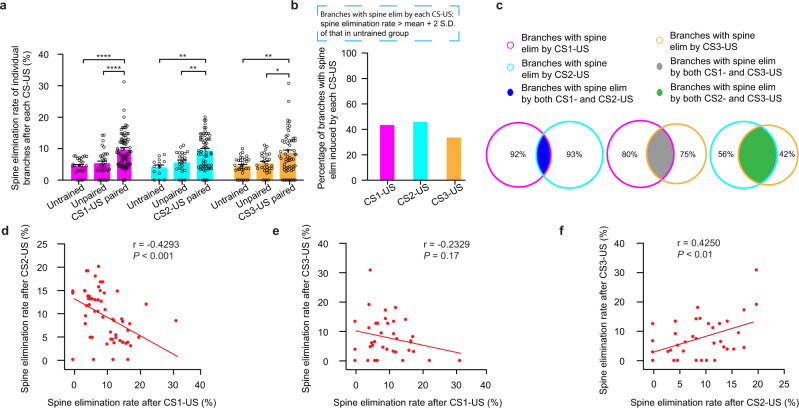
Distribution of spine elimination induced by different CS-US pairings on individual dendrites. **a** Comparisons of spine elimination rates on individual dendritic branches between mice subjected to each CS-US pairing and untrained or unpaired control mice (CS1: 1 kHz; CS2: 10 kHz; CS3: 5 kHz; CS1-US pairing vs. untrained or unpaired, *P* < 0.0001; *n* = 78, 28, and 34 branches, respectively. CS2-US pairing vs. untrained or unpaired, *P* = 0.0014 or *P* = 0.0075; *n* = 60, 14, and 24 branches respectively. CS3-US pairing vs. untrained or unpaired, *P* = 0.0024 and *P* = 0.0233, respectively; *n* = 58, 32, and 28 branches, respectively; Mann–Whitney *U* test). **b** After each CS-US pairing, ~40% of individual dendritic branches showed spine elimination rate higher than the mean plus two times the standard deviation of that in untrained control mice. **c** Percentage of overlap between branches with spine elimination induced by any two different CS-US pairing. The percentage in the non-overlapping area indicates the proportion of non-overlapping branches versus branches with spine elimination induced by each of different CS-US pairings. **d** The rate of spine elimination rate on individual branches after CS2-US pairing was inversely correlated with that after CS1-US pairing (*P* = 0.0006, Pearson’s correlation; *n* = 60 branches). **e** The rate of spine elimination on individual branches after CS3-US pairing was not correlated with that after CS1-US pairing (*P* = 0.1717, Pearson’s correlation; *n* = 36 branches). **f** The rate of spine elimination on individual branches after CS3-US pairing was positively correlated with that after CS2-US pairing (*P* = 0.0098, Pearson’s correlation). Error bars, ±S.E.M. All statistical tests were performed two-sided. * *P* < 0.05; ** *P* < 0.01; **** *P* < 0.0001.

We next investigated whether spine elimination induced by different CS-US parings is intermingled or segregated on individual dendritic branches of layer 5 pyramidal neurons (Fig. 3a, b; CS1: 1 kHz, CS2: 10 kHz, CS3: 5 kHz; average branch length: CS1-US pairing, 94.0 ± 3.1 µm, *n* = 78 branches from 33 mice; CS2-US pairing, 90.3 ± 3.5 µm, *n* = 60 branches from 28 mice; CS3-US pairing, 90.9 ± 5.4 um, *n* = 36 branches from 14 mice). As expected, the average rate of spine elimination on individual dendritic branches after each CS-US pairing was significantly higher than that in untrained or unpaired control mice (Fig. 4a; spine elimination rate: CS1-US pairing, 9.5 ± 0.6%; untrained, 4.5 ± 0.5%; unpaired, 5.4 ± 0.7%; Pairing vs. untrained or unpaired, *P* < 0.0001; *n* = 78, 28 and 34 branches respectively. CS2-US pairing, 9.3 ± 0.7%; untrained, 4.1 ± 0.7%; unpaired, 5.8 ± 0.7%; Pairing vs. untrained or unpaired, *P* < 0.01; *n* = 60, 14, and 24 branches respectively. CS3-US pairing, 8.6 ± 0.9%; untrained, 4.5 ± 0.5%; unpaired, 5.3 ± 0.6%; Pairing vs. untrained or unpaired, *P* < 0.01 or *P* < 0.05, respectively; *n* = 58, 32 and 28 branches respectively). Importantly, each CS-US pairing induced spine elimination only on a subset of apical tuft branches when compared to that in untrained control mice (Fig. 4b and Supplementary Fig. 7; CS1-US pairing, ~42%; CS2-US pairing, ~47%; CS3-US pairing, ~36%).

In mice subjected to two different CS-US pairings, only a small fraction (~8%) of dendritic branches with spine elimination induced by CS1-US pairing showed spine elimination induced by CS2-US pairing (Fig. 4c; CS1: 1 kHz, CS2: 10 kHz; total 60 branches after CS1-US and CS2-US pairings). Assuming that CS2-US pairing induces spine elimination randomly on branches with or without spine elimination induced by CS1-US pairing, we estimated that ~46% (instead of 8%) of branches with spine elimination induced by CS1-US pairing would show spine elimination induced by CS2-US pairing (Supplementary Fig. 8a). The rate of spine elimination after CS2-US pairing was inversely correlated with that after CS1-US pairing at the dendritic branch level (Figs. 3b, 4d, CS1: 1 kHz, CS2: 10 kHz; *P* < 0.001; Supplementary Fig. 9, CS1: 10 kHz, CS2: 5 kHz, *P* < 0.01; Pearson’s correlation). Because the majority of layer 5 pyramidal neurons show increased spine elimination induced by both CS1-US and CS2-US pairings (Fig. 3e), these results suggest that two different CS-US pairings induce spine elimination mainly on separate branches of the same neuron.

In mice subjected to three different CS-US pairings, we found that the rate of spine elimination after CS3-US pairing showed no correlation with that induced by CS1-US pairing (Fig. 4e; *P* = 0.17, Pearson’s correlation) and a positive correlation with that induced by CS2-US pairing (Fig. 4f; *P* < 0.01, Pearson’s correlation). Compared with a small percent of dendritic branches with spine elimination induced by both CS1-US and CS2-US pairings, a significantly larger fraction of dendritic branches with spine elimination after CS1-US pairing (20%, 15 branches) or after CS2-US pairing (~44%, 16 branches) also showed spine elimination induced by CS3-US pairing respectively (Fig. 4c and Supplementary Fig. 8b; CS1: 1 kHz, CS2: 10 kHz, CS3: 5 kHz; ~20% vs. 8%, *P* = 0.25; ~44% vs. 8%, *P* < 0.01; Supplementary Fig. 10; CS1: 5 kHz, CS2: 10 kHz, CS3: 1 kHz; ~25% vs. 0%, *P* = 0.13; ~50% vs. 0%, *P* < 0.05; chi square test). Thus, in mice subjected to three different CS-US pairings, branch-specific spine elimination starts to breakdown: a substantial fraction of individual dendritic branches undergo spine elimination induced by both CS2-US and CS3-US pairings.

### Segregated distribution of spine elimination is associated with CS-specific spine formation and neuronal activity increase after fear extinction

The above results show that two different CS-US pairings induce spine elimination mainly on separate dendritic branches of individual neurons, whereas three different CS-US pairings induce spine elimination often on the same dendritic branches. Because mice subjected to two or three different CS-US pairings exhibit CS-specific or generalized fear extinction respectively, these results raise the possibility that segregated or intermingled dendritic distribution of spine elimination induced by different CS-US pairings may be linked to specific or generalized modification of individual neurons by fear extinction stimuli. To test this possibility, we first examined spine formation as well as neuronal responses after fear extinction in mice subjected to two different CS-US pairings.

**Fig. 5:**
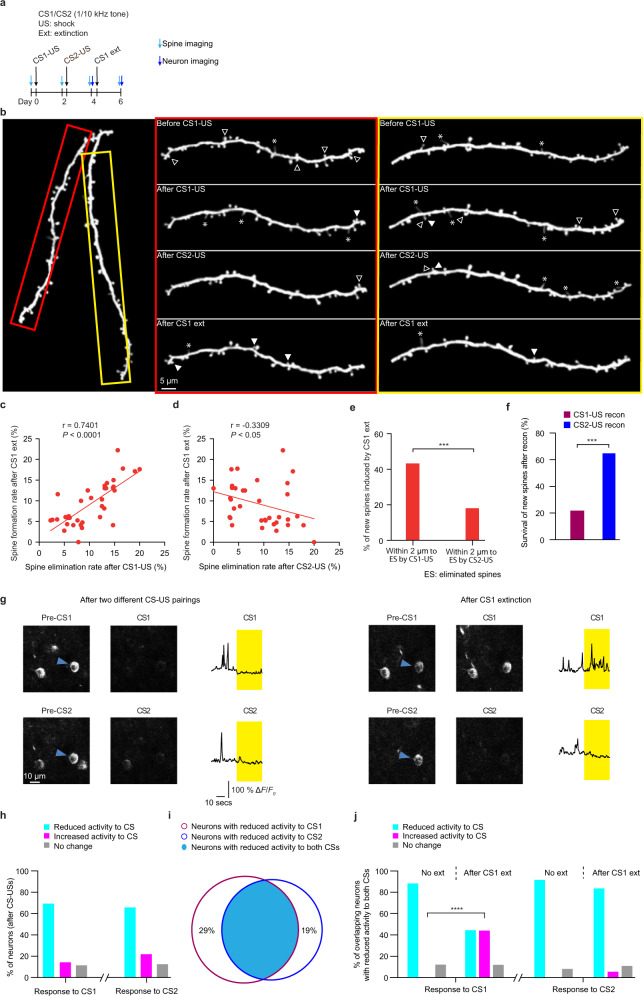
Fear extinction induces CS-specific spine formation and neuronal activity increase in response to CS when two different CS-US pairings induce spine elimination on separate dendritic branches. **a** Experimental design to examine spine remodeling and somatic Ca^2+^ activity in mice subjected to two different CS-USs followed by CS1 extinction (CS1: 1 kHz; CS2: 10 kHz). **b** Representative images of spine remodeling induced by two different CS-US pairings followed by CS1 extinction on individual dendritic branches in YFP-H mice. The hollow triangles indicate spines eliminated in the succeeding image. The solid triangles indicate newly-formed spines when compared to the preceding image. The asterisks indicate filopodia. Experiments were repeated independently on 36 branches in 8 mice with similar results. The rate of spine formation on individual branches after CS1 extinction was positively correlated with the rate of spine elimination after CS1-US pairing (**c**) and inversely correlated with the rate of spine elimination after CS2-US pairing (**d**) (*P* < 0.0001 and *P* = 0.0487, respectively, Pearson’s correlation; *n* = 36 branches). **e** Majority of newly-formed spines after CS1 extinction were located within 2 µm distance to spines eliminated after CS1-US pairing, but not after CS2-US pairing (*P* = 0.0002, chi-square test; *n* = 91 spines from 8 mice). **f** The survival rate of newly-formed spines induced by CS1 extinction was lower after reconditioning by CS1-US pairing than after reconditioning by CS2-US pairing (*P* = 0.0002, chi-square test; *n* = 36 and 40 spines from 6 mice, respectively). **g** Left panel: Representative images of somatic activity of layer 5 pyramidal neurons in GCaMP6S line 3 mice after CS1-US and CS2-US pairings; The blue triangle indicates the soma with reduced activity to both CS1 and CS2 as compared to the pre-CS period after two different CS-US pairings. Somatic Ca^2+^ changes in this cell were measured by Δ*F*/*F*_0_. Right panel: Representative images of somatic activity in response to CS1 or CS2 after CS1 extinction. The labeled neuron with reduced response to both CSs in the (left panel) showed increased activity to CS1 but not CS2 after CS1 extinction. Experiments were repeated independently in 189 somas in 10 mice with similar results. **h** After CS1-US or CS2-US pairings, the majority of neurons exhibited reduced somatic Ca^2+^ activity in response to CS1 or CS2 as compared to pre-CS period (*n* = 189 somas from 10 mice). **i** Percentage of overlap between neurons with reduced activity to two different CSs. The percentage in the non-overlapping area indicates the proportion of non-overlapping neurons relative to neurons with reduced activity to each CS. **j** After CS1 extinction, a larger percentage of overlapping neurons with reduced activity to both CSs showed increased activity in response to CS1 when compared to without extinction (*P* < 0.0001, chi-square test; *n* = 66 and 25 somas in extinction and no extinction group, respectively). The percentage of overlapping neurons exhibiting increased activity in response to CS2 after CS1 extinction was comparable to that after no extinction (*P* = 0.2081, chi-square test). All statistical tests were performed two-sided. *** *P* < 0.001; **** *P* < 0.0001.

In mice subjected to two different CS-US pairings (Fig. 5a), the rate of spine formation after CS1 extinction was positively correlated with the rate of spine elimination after CS1-US pairing (Fig. 5b, c and Supplementary Fig. 11a; CS1: 1 kHz, CS2: 10 kHz; *n* = 36 branches; *P* < 0.0001, Pearson’s correlation) and inversely correlated with that after CS2-US pairing at the level of individual branches (Fig. 5d; *P* < 0.05, Pearson’s correlation). Previous studies have shown that many new spines formed after fear extinction are located near eliminated spines induced by fear conditioning^48^. Consistently, we found that newly-formed spines after CS1 extinction tended to be located within 2 µm to spines eliminated after CS1-US pairing, rather than to spines eliminated after CS2-US pairing (Fig. 5e and Supplementary Fig. 11b; ~42.9% vs. 17.6%, *P* < 0.001, chi-square test; *n* = 91 spines from 8 mice). In addition, these new spines were preferentially eliminated after reconditioning by CS1-US pairing, but not CS2-US pairing (Fig. 5f; survival rate: ~22.2% vs. 65.0%; *P* < 0.001, chi square test; *n* = 36 and 40 spines from 6 mice, respectively). Taken together, these results indicate that when two different CS-USs induce CS- and dendritic branch-specific spine elimination, subsequent fear extinction induces CS- and branch-specific spine formation. Furthermore, new spines induced by CS1 extinction may be functionally similar to those spines eliminated after CS1-US pairing as both are eliminated by the same CS-US stimuli that were presented during fear conditioning or re-conditioning.

When Ca^2+^ imaging was performed to measure activity changes of layer 5 pyramidal neurons in the motor cortex of mice receiving two different CS-USs (Fig. 5g), ~71% of neurons with reduced activity in response to CS1 also showed reduced activity in response to CS2 (Fig. 5h, i; n = 189 somas from 10 mice after CS1-US and CS2-US pairings). After CS1 extinction, neurons with reduced activity by both CS1-US and CS2-US pairings increased activity specifically in response to CS1, but not CS2, during the recall test (Fig. 5j, P < 0.0001, chi square test). Together with the findings in Fig. 4a-d, these results suggest that when spine elimination induced by two different CS-US pairings are segregated on different dendritic branches of layer 5 pyramidal neurons, these neurons undergo CS-specific spine formation and activity increase after fear extinction.

### Intermingled dendritic distribution of spine elimination is associated with CS-nonspecific spine formation and neuronal activity increase by fear extinction

**Fig. 6:**
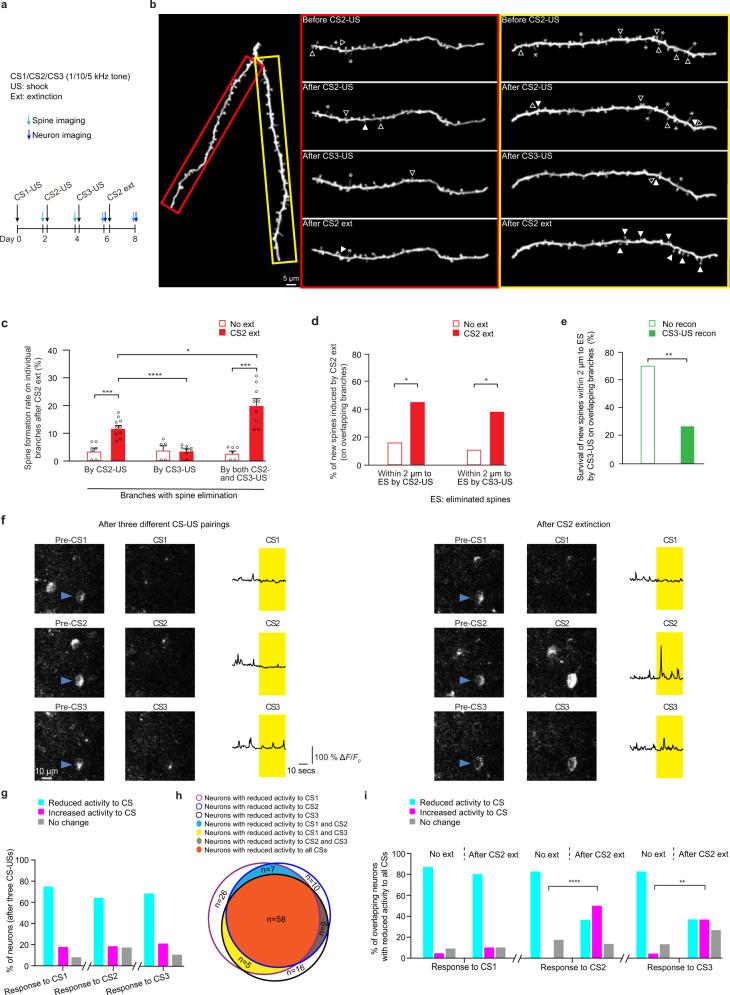
Fear extinction induces CS-nonspecific spine formation and neuronal activity increase in response to CS when spine elimination induced by three different CS-US pairings occurs on the same dendritic branches. **a** Experimental design to examine spine remodeling and somatic Ca^2+^ activity in mice subjected to three different CS-USs followed by CS2 extinction respectively (CS1: 1 kHz; CS2: 10 kHz; CS3: 5 kHz). **b** Representative images of spine remodeling induced by CS2-US and CS3-US pairings followed by CS2 extinction on individual dendritic branches in YFP-H line mice. The hollow triangles indicate spines eliminated in the succeeding image. The solid triangles indicate newly-formed spines when compared to the preceding image. The asterisks indicate filopodia. Experiments were repeated independently on 27 branches in 7 mice with similar results. **c** Comparisons of spine formation rates after CS2 extinction on branches with spine elimination induced by one or more CS-US pairings (spine formation rate after CS2 extinction on branches with spine elimination induced by only CS2-US pairing vs. by only CS3-US pairing or by both CS2-US and CS3-US pairing, *P* < 0.0001 or *P* < 0.0108, respectively; spine formation rate after CS2 extinction vs. no extinction on branches with spine elimination induced by only CS2-US pairing or by both CS2-US and CS3-US pairing, *P* = 0.0002 or *P* = 0.0004, respectively; Mann–Whitney *U* test; *n* = 10, 8, and 9 branches with spine elimination induced by only CS2-US or by only CS3-US or by both CS2-US and CS3-US in CS2 extinction group; *n* = 6, 5, and 6 branches with spine elimination induced by only CS2-US pairing or by only CS3-US pairing or by both CS2-US and CS3-US pairings in no extinction group). **d** On branches with spine elimination induced by both CS2-US and CS3-US pairings, a larger percentage of new spines induced by CS2 extinction were located within 2 µm to spines eliminated by CS2-US pairing or by CS3-US pairing as compared to no extinction condition (*P* = 0.0234 or 0.0252, respectively, chi-square test; *n* = 58 and 19 newly-formed spines from 7 and 5 mice in extinction and no extinction groups respectively). **e** On branches with spine elimination induced by both CS2-US pairing and CS3-US pairings, CS3-US reconditioning reduces the survival rate of new spines that were formed after CS2 extinction and located within 2 µm to spines eliminated prior CS3-US pairing (*P* = 0.0019, compared to no reconditioning group; chi square test; *n* = 30 and 23 newly-formed spines from 8 and 4 mice in reconditioning and no reconditioning groups respectively). **f** Left panel: Representative images of somatic activity of layer 5 pyramidal neurons in GCaMP6S line 3 mice after three different CS-US pairings. The blue triangle indicates the soma with reduced activity to all CSs as compared to the pre-CS period after three CS-US pairings. Right panel: Representative images of somatic activity in response to CS1, CS2, or CS3 after CS2 extinction. The labeled neuron with reduced response to all CSs in (left panel) showed increased activity to CS2 and CS3, but not CS1, after CS2 extinction. Experiments were repeated independently in 130 somas in 7 mice with similar results. **g** After three different CS-US pairings, the majority of layer 5 pyramidal neurons exhibited reduced somatic Ca^2+^ activity in response to CS1, CS2, or CS3 as compared to the pre-CS period (*n* = 130 somas from 7 mice). h. Overlap among neurons with reduced activity to different CSs. The number in the overlapping or non-overlapping area indicates the number of neurons with reduced activity to different CSs. **i** After CS2 extinction, a larger percentage of overlapping neurons with reduced activity to all CSs showed increased activity to CS2 and CS3 when compared to that without no extinction (CS2, *P* < 0.0001, chi-square test; CS3, *P* < 0.0053; *n* = 30 and 23 somas in extinction and no extinction groups respectively). The percentage of these overlapping neurons exhibiting increased activity to CS1 after CS2 extinction was comparable to that without extinction (*P* = 0.4401, chi-square test). Error bars, ±S.E.M. All statistical tests were performed two-sided. * *P* < 0.05; ** *P* < 0.01; *** *P* < 0.001; **** *P* < 0.0001.

As shown in Fig. 4, in mice subjected to three different CS-US pairings (CS1: 1 kHz, CS2: 10 kHz, CS3: 5 kHz), spine elimination induced by CS2-US and CS3-US pairings often occurs on the same dendritic branches (Fig. 4c, e, f). After CS2 extinction, we found that the spine formation rate was significantly higher on branches with spine elimination induced by either CS2-US pairing or by both CS2-US and CS3-US pairings, when compared to that on branches with spine elimination induced by only CS3-US pairing or no extinction group (Fig. 6a–c; *P* < 0.0001 and *P* < 0.001 respectively). Notably, the spine formation rate was significantly higher on branches with spine elimination induced by both CS2-US and CS3-US pairings than by only CS2-US (Fig. 6c and Supplementary 12; *P* < 0.05). Similar results were also observed after CS3 extinction (Supplementary Fig. 13). Furthermore, on those branches with spine elimination induced by both CS2-US and CS3-US pairings, ~44.8% and ~37.9% of newly-formed spines induced by CS2 extinction were located within 2 µm to spines eliminated by CS2-US pairing or by CS3-US pairing, respectively (Fig. 6d; *n* = 58 newly-formed spines from 7 mice). Without extinction, only a small fraction (15.8% and 10.5%) of newly-formed spines were located within 2 µm of spines eliminated by CS2-US pairing or by CS3-US pairing (Fig. 6d; *P* < 0.05 respectively, chi-square test, compared to that in the extinction group; *n* = 19 newly-formed spines from 5 mice).

In addition, we identified new spines induced by CS2 extinction and examined their survival rate after reconditioning the mice with CS3-US pairing (Fig. 6e). After CS3-US reconditioning, the survival rate of new spines located within 2 µm to spines eliminated after the initial CS3-US pairing on branches with spine elimination induced by both CS2-US and CS3-US pairings was significantly lower, when compared to no reconditioning controls (Fig. 6e; 26.7% versus 69.6%, *P* < 0.01, chi-square test; *n* = 30 and 23 newly-formed spines from 8 and 4 mice respectively). By contrast, new spines located beyond 2 µm to spines eliminated after the initial CS3-US pairing on branches with spine elimination induced by both CS2-US and CS3-US pairings were not preferentially eliminated after CS3-US reconditioning (Supplementary Fig. 14a; survival rate: 65.6% versus 73.1%, *P* = 0.54; *n* = 32 and 26 newly-formed spines from 8 and 4 mice in reconditioning and no reconditioning groups respectively). Furthermore, on branches with spine elimination induced by only CS2-US pairing, new spines induced by CS2 extinction were not preferentially eliminated after CS3-US reconditioning (Supplementary Fig. 14b; survival rate: 63.2%, *P* = 0.31, compared to 74.3% in no reconditioning group, chi-square test; *n* = 38 and 35 newly-formed spines in four mice, respectively). Together, these results suggest that on branches with spine elimination induced by both CS2-US and CS3-US pairings, newly-formed spines induced by CS2 extinction and close to spines eliminated by CS3-US pairing may be functionally similar to spines eliminated by CS3-US pairing. They also suggest that the co-existence of spine elimination induced by different CS-US pairings on the same dendritic branches results in spine formation by subsequent fear extinction in a CS-generalized manner.

Consistent with the findings above, we found that in mice receiving three different CS-USs, a large fraction of layer 5 pyramidal neurons with reduced somatic Ca^2+^ activity in response to CS1 (~66%) or CS2 (~80%) showed reduced activity in response to CS3 (Fig. 6g, h; *n* = 130 somas from 7 mice for three different CS-USs). Importantly, after CS2 extinction, neurons with reduced somatic Ca^2+^ activity by all three CS-US pairings increased activity to both CS2 and CS3, but not CS1, during the recall test (Fig. 6f, i). Moreover, the increased somatic Ca^2+^ activity in response to all CSs during the recall test was observed after CS3 extinction (Supplementary Fig. 15), and the percentage of branches with spine elimination induced by CS1-US/CS2-US pairings, CS1-US/CS3-US pairings and CS2/US/CS3-US pairing correlated with the generalized reduction of freezing response after CS2 or CS3 extinction (Supplementary Fig. 16). Together, these results suggest that individual neurons undergo CS non-specific modification of spine formation and activity increase after fear extinction when spine elimination induced by different CS-US pairings co-exists on the same dendritic branches of individual neurons.

### SST interneuron activity regulates the dendritic distribution of spine elimination induced by different CS-US pairings and subsequent changes induced by fear extinction

Previous studies have shown that somatostatin (SST) expressing interneurons (INs) target apical dendrites of pyramidal neurons and is important for regulating dendritic activity and synaptic plasticity^52–58^. To better understand the dendritic distribution of spine elimination induced by different CS-US pairings and its relationship with specific or generalized fear extinction, we altered the activity of SST INs and examined the impact of such manipulation on spine remodeling and behavioral outcome in mice subjected to fear conditioning and extinction.

**Fig. 7:**
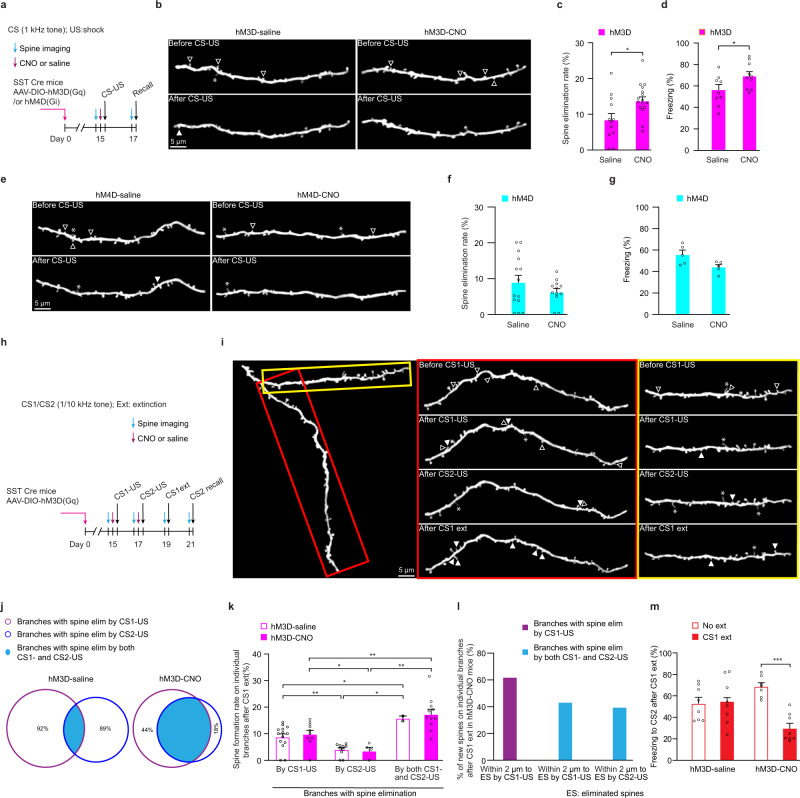
SST interneuron activity regulates dendritic distribution of spine elimination induced by different CS-US pairings and subsequent changes induced by fear extinction. **a** Experimental design to examine the regulation of fear conditioning-induced spine elimination by SST INs (CS1: 1 kHz). **b** Representative images of spine remodeling on apical dendrites induced by fear conditioning in YFP-H line mice crossed with SST Cre mice which were infected with hM3D(Gq) and injected with either CNO or saline. Experiments were repeated independently on 16 branches in 5 hM3D(Gq)-CNO mice and 12 branches in 5 hM3D(Gq)-saline mice with similar results. The hollow triangles indicate spines eliminated in the succeeding image. The solid triangles indicate newly-formed spines when compared to preceding image. The asterisks indicate filopodia. **c** After fear conditioning, the spine elimination rate on individual branches was significantly higher in the hM3D(Gq)-CNO group than the hM3D(Gq)-saline control group (*P* = 0.016, unpaired *t* test; *n* = 12 and 16 branches in hM3D(Gq)-saline and hM3D(Gq)-CNO groups respectively). **d** The freezing response was significantly higher in the hM3D(Gq)-CNO group than the hM3D(Gq)-saline control group (*P* = 0.0349, unpaired t-test; *n* = 9 and 10 mice in hM3D(Gq)-saline and hM3D(Gq)-CNO groups, respectively). **e** Representative images of spine remodeling on apical dendrites induced by fear conditioning in YFP-H-line mice crossed with SST Cre mice which were infected with hM4D(Gq) and injected with CNO or saline. Experiments were repeated independently on 12 branches in 4 hM4D(Gi)-saline mice and 14 branches in 4 hM4D(Gi)-saline mice with similar results. **f** After fear conditioning, the spine elimination rate of individual branches was slightly but not significantly lower in the hM4D(Gi)-CNO group than the hM4D(Gi)-saline group (*P* = 0.2502, unpaired *t* test; *n* = 14 and 12 branches in hM4D(Gi)-saline and hM4D(Gi)-CNO groups respectively). **g** The freezing response was slightly lower in the hM4D(Gi)-CNO group than the hM4D(Gi)-saline group (*P* = 0.0952, Mann–Whitney *U* test; *n* = 5 mice for each group). **h** Experimental design (CS1: 1 kHz; CS2: 10 kHz). **i** Representative images of spine remodeling induced by two different CS-US pairings followed by CS1 extinction on individual dendritic branches in hM3D(Gq)-CNO mice. Experiments were repeated independently on 11 branches in 7 mice with similar results. **j** Percentage of overlap between branches with spine elimination induced by two different CS-US pairing in hM3D(Gq)-saline and hM3D(Gq)-CNO mice. The percentage in the non-overlapping area indicates the proportion of non-overlapping branches relative to branches with spine elimination induced by each of different CS-US pairings. **k** After CS1 extinction, the spine formation rate was significantly higher on branches with spine elimination induced by both CS1-US and CS2-US pairings when compared to that on branches with spine elimination induced by only CS1-US pairing or by only CS2-US pairing in both hM3D(Gq)-CNO and hM3D(Gq)-saline groups (hM3D(Gq)-CNO group: both CS-US pairings vs. CS1-US or CS2-US pairing, *P* = 0.005 or *P* = 0.0015, respectively; CS1-US vs. CS2-US pairing, *P* = 0.017; Mann–Whitney *U* test; *n* = 11, 10, and 4 branches with spine elimination induced by both CS1 and CS2-US pairings or by only CS1-US pairing or by only CS2-US pairing; hM3D(Gq)-saline group: both CS-US pairings vs. CS1-US or CS2-US pairing, *P* = 0.0167 or *P* = 0.0303; CS1-US vs. CS2-US pairing, *P* = 0.0025; Mann–Whitney U test; n = 2, 14 and 10 branches with spine elimination induced by both CS1-US and CS2-US pairings or by only CS1-US pairing or by only CS2-US pairing in hM3D(Gq)-saline group). **l** On branches with spine elimination induced by both CS1-US and CS2-US pairings, a large percentage of newly-formed spines after CS1 extinction were located within 2 µm to spines eliminated by CS1-US pairing or by CS2-US pairing respectively in hM3D(Gq)-CNO group (*n* = 54 newly-formed spines from 7 mice). **m** After CS1 extinction, the freezing response to CS2 in the hM3D(Gq)-CNO group was significantly lower when compared to that without extinction (*P* = 0.0003, Mann–Whitney *U* test; n = 7-8 mice for each group). Error bars, ±S.E.M. All statistical tests were performed two-sided. *P < 0.05; **P < 0.01; ***P < 0.001.

In order to manipulate the activity of SST INs in the motor cortex, we used the pharmacogenetic approach involving Designer Receptor Exclusively Activated by Designer Drug (DREADD) and Clozapine-N-oxide (CNO)^59^. In this experiment, SST neurons in SST Cre mice were infected with AAV viruses to express either the activating hM3D(Gq) or inhibiting hM4D(Gi) form of DREADD (Fig. 7a and Supplementary Fig. 17a)^60^, and CNO was administered to increase or decrease the activity of SST INs 30 min before fear conditioning (Supplementary Fig. 17b, c). When compared to saline-injected control, activation of SST INs by CNO injection in the hM3D(Gq)-expressing mice before CS-US pairing increased the spine elimination rate on individual branches and freezing response (Fig. 7b–d; CS: 1 kHz; spine elimination rate: hM3D(Gq)-CNO, 14.0 ± 1.2%; hM3D(Gq)-saline, 8.6 ± 1.8%; *P* < 0.05; *n* = 16 and 12 branches respectively), while inactivation of SST INs by CNO injection in the hM4D(Gi)-expressing mice slightly decreased spine elimination rate and freezing response (Fig. 7e–g; spine elimination rate: hM4D(Gi)-CNO, 6.2 ± 1.0%; hM4D(Gi)-saline, 8.9 ± 2.0%; *P* = 0.25; *n* = 12 and 14 branches).

We next examined whether activation of SST INs affected the occurrence of spine elimination induced by two different CS-US pairings on the same dendritic branches as well as subsequent fear extinction generalization (Fig. 7h; CS1: 1 kHz, CS2: 10 kHz). Notably, ~56% of dendritic branches with spine elimination induced by CS1-US pairing showed spine elimination induced by CS2-US pairing in hM3D(Gq)-CNO mice (Fig. 7i, j; spine elimination rate by CS2-US, 11.6 ± 2.1%; *P* < 0.01, chi-square test; total 18 branches from 5 mice after CS1-US and CS2-US pairings). In contrast and as expected, ~8% of dendritic branches with spine elimination induced by CS1-US pairing showed spine elimination induced by CS2-US pairing in hM3D(Gq)-saline control mice (Fig. 7j; spine elimination rate by CS2-US, 4.8 ± 1.2%; total 28 branches from 5 mice after CS1-US and CS2-US pairings). After CS1 extinction, the spine formation rate on branches with spine elimination induced by both CS1-US and CS2-US pairings was significantly higher than that on branches with spine elimination induced by only CS1-US pairing in both hM3D(Gq)-CNO and hM3D(Gq)-saline mice (Fig. 7k; *P* < 0.01). Furthermore, a large percentage of newly-formed spines on branches with spine elimination induced by CS1-US and CS2-US pairings were located within 2 µm to spines eliminated by CS1-US pairing or by CS2-US pairing (Fig. 7l and Supplementary Fig. 18; *n* = 54 and 9 newly-formed spines from 7 and 5 mice in hM3D(Gq)-CNO and hM3D(Gq)-saline groups respectively). In addition, CS1 extinction significantly decreased the freezing response to CS2 compared to that without extinction in hM3D(Gq)-CNO mice (Fig. 7m; *P* < 0.001). Together, these results indicate that the activation of SST INs increases the occurrence of spine elimination induced by different CS-US pairings on the same dendritic branches, as well as subsequent CS non-specific spine formation and the generalization of fear extinction.

## Discussion

Memory generalization is an important feature of information storage in the brain, but the underlying mechanisms remain unclear. Previous studies have shown that when memories are encoded by overlapping neuronal populations, modification of one memory is often, although not always, generalized to the other memories^10–18,61^. By investigating dendritic spine remodeling, neuronal activity, and freezing behaviors in response to fear conditioning and extinction, our findings show that the overlap of neurons encoding different fear memories does not necessarily lead to fear extinction generalization. Instead, specific or generalized fear extinction depends on whether synaptic structural remodeling associated with fear memories segregates or co-exists on individual dendritic branches. These results provide important insights into how the distribution of synaptic plasticity within complicated neuronal circuits underlies memory generalization.

Our results show that fear conditioning with two different CS-USs induce spine elimination mainly on different dendritic branches while spine elimination induced by fear conditioning with three CS-USs often occurs on the same dendritic branches in the motor cortex. We presented several lines of evidence supporting that this segregation or co-existence of spine elimination induced by different CS-US pairings on dendritic branches is critical for fear extinction specificity or generalization. First, when mice were subjected to two different CS-USs, subsequent fear extinction caused spine formation in a CS-specific manner and resulted in the CS-specific reversal of neuronal response and freezing behavior induced by fear conditioning. Second, when spine elimination induced by different CS-US pairings co-existed on the same dendritic branches in mice subjected to three CS-USs, fear extinction with one CS-induced spine formation on those branches in a CS-nonspecific manner and resulted in generalized modification of neuronal and behavioral responses. Third, increasing the occurrence of spine elimination induced by different CS-US parings on the same dendritic branches by manipulating SST INs activity in mice subjected to two different CS-USs facilitated fear extinction generalization. Together, these findings suggest that specific or generalized fear extinction depends on whether spine elimination induced by different CS-US pairings is segregated or co-exists on dendritic branches.

Previous studies have shown that neuronal activity or experience can trigger synaptic plasticity within small segments of dendritic branches or a subset of individual dendritic branches^31–33,62–66^. In the present study, fear conditioning with different CS-USs induces spine elimination on a subset of dendritic branches of individual layer 5 pyramidal neurons in the motor cortex. These findings support a view that individual dendritic branches, rather than individual layer 5 pyramidal neurons, act as independent units for synaptic plasticity associated with memory storage^66,67^. Our results further suggest that such a dendritic branch-specific mode for memory storage involves the activity of SST INs and has the advantage of reducing generalized modification by new experience. The precise mechanisms underlying this function of SST INs need further investigation. GABAergic inhibition has been reported to exert compartmentalized control over Ca^2+^ signals within dendritic spines and to promote long-term depotentiation and spine shrinkage^53,68^. Therefore, SST IN activation may increase fear conditioning-induced spine elimination by affecting Ca^2+^ signals of spines on individual dendritic branches. In addition, SST INs are known to inhibit other inhibitory neurons such as neurogliaform cells and PV neurons^69,70^. It is thus also possible that SST IN activity may reduce the activity of other inhibitory neurons to increase the excitability and fear conditioning-induced spine elimination on layer 5 pyramidal dendrites.

Our results indicate that fear extinction leads to non-specific spine formation when spines eliminated by different CS-US pairings are located on the same dendritic branches. Previous studies have reported that long-term potentiation (LTP) elicited at specific synapses can facilitate the potentiation at other synapses on the same but not different dendritic branches^66,71^. LTP at specific spines may affect potentiation at other spines on the same dendritic branches via Ca^2+^-dependent RAS activity or active RhoA diffusing from activated spines^72,73^. It is possible that synaptic activities related to one CS induced by fear extinction may trigger such signaling molecules to diffuse along dendritic branches and facilitate the formation of new spines related to spines eliminated by different CS-US pairings. Further studies of how Ca^2+^-dependent downstream signaling facilitates synapse formation on the same dendritic branches would help to understand the molecular basis for the generalization of fear memory extinction.

Our results indicate that inactivating the auditory cortex before CS2 extinction prevented the reduction of freezing response to CS2 but not CS3. On the other hand, inactivating the motor cortex before CS2 extinction prevented the reduction of freezing response to CS2 and CS3. The reasons for the different effects of inactivating cortical regions on fear extinction generalization are not clear. Previous studies have shown that the inactivation of auditory cortex does not prevent fear generalization^74^, while increasing CREB level in auditory thalamus enhances fear generalization^75^. It is possible that the auditory cortex is involved in cue-specific fear extinction whereas the thalamic pathway transmitting CS information to the brain regions such as the amygdala and motor cortex contributes to the generalization of fear extinction. Such circuit connectivity could in principle explain why inactivating motor cortex affects fear extinction generalization but inactivating auditory cortex only affects fear extinction. It would be interesting to investigate these possibilities in the future.

It is important to note that our study here focuses on how spine plasticity on apical dendritic branches of layer 5 pyramidal neurons in the motor cortex is involved in the generalization of fear extinction. Previous studies have shown that multiple brain regions including the medial prefrontal cortex, lateral amygdala, medial prefrontal cortex, nucleus reuniens, hippocampus, and thalamus are important for fear generalization^74,76,77^. It would be of interest to investigate whether the generalization of fear extinction involves synaptic plasticity at the dendritic branch or neuronal level in other brain regions than the motor cortex. Furthermore, although spines eliminated by fear conditioning and formed by fear extinction likely contribute significantly to the changes of neuronal activity and fear responses, changes of neuronal excitability and inhibition are also likely important in the processes^12,13,78,79^. Further studies are needed to perturb synaptic plasticity specifically and determine the consequence of such manipulation on changes and fear memory generalization.

## Methods

### Experimental animals

In all experiments, male and female mice at age of 28–30 days were used unless otherwise specified. Mice expressing YFP (H line) in cortical pyramidal neurons, SST Cre mice (Ssttm2.1(cre)Zjh/J) and C57BL/J mice were obtained from the Jackson Laboratory. Mice expressing GCaMP6 (GCaMP6S Line 3) in cortical pyramidal neurons were generated in the transgenic facility at NYU medical center^80^. Mice were group-housed (3–5 per cage) in a 12:12 light/dark cycle (lights on at 07:00 a.m.) at 22 ± 2 °C (60–65% humidity). All experiments were performed in accordance with Institutional Animal Care and Use Committees (IACUC) of New York University School of Medicine and Peking University Shenzhen Graduate School.

### Behavioral training and testing

Fear conditioning and extinction were performed in a cage within a sound-attenuating box (Coulbourn Instruments). Behavior was recorded by low-light video cameras. Stimulus presentation was automated by using Actimetrics FreezeFrame 4.0 software (Coulbourn Instruments).

Fear conditioning was performed in a training cage equipped with stainless-steel shocking grids connected to a precision feedback current-regulated shocker. The inner walls and floor of the cage were covered with paper, or plastic film. The ethanol, pinesol or lemon scent was put on the floor of the cage. Mice were habituated for 2 min on shocking grids and then received seven presentations of a 30-s auditory tone (80 dB, cage enclosed) co-terminating with a 2-s, 0.6-mA footshock. The intertrial interval was 90 s. One minute after training, mice were returned to their home cages. For the unpaired group, mice received seven presentations of auditory tone and shock that were separated by random intervals of 20–40 s. For the untrained group, mice were habituated in the training cage without tone or shock presentations.

Auditory tones of 1, 2.5, 5, and 10 kHz were used as different CSs. Previous studies have shown that tones at these frequencies evoke freezing responses after fear conditioning training, although mice are more sensitive for tones at higher frequencies^43,48,81^. In mice subjected to two different CS-US pairings, 1 kHz (CS1) and 10 kHz (CS2) tones, 10 kHz (CS1) and 5 kHz (CS2) tones or 2.5 kHz (CS1) and 1 kHz (CS2) tones were used as CS1 and CS2 respectively to examine whether fear extinction is specific when two CS stimuli differ in frequency. In mice subjected to three different CS-US pairings, 1,10 and 5 kHz tones were used to examine whether the increase in the number of stimuli affected fear extinction specificity. To rule out that CS stimuli are being perceived differently and that the sequence the stimuli presented in matters, 1 kHz (CS1), 10 kHz (CS2) and 5 kHz (CS3) tones, or 5 kHz (CS1), 10 kHz (CS2) and 1 kHz (CS3) tones were used as CS1, CS2, and CS3, respectively. When mice were subjected to fear conditioning with 2–4 different CS-US pairings, each CS-US pairing was separated by 2 days from each other and was performed in a cage with a different context by changing the covering of the walls and floor of the cage and the scent put in the cage.

Fear extinction was performed in a cage with a context different from the cage used in fear conditioning, by changing the covering of the walls and floor of the cage. No scent was put in the cage. The cage was equipped with non-shocking grids. Mice were habituated for 2 min and received fifteen presentations of the same auditory tone that was used in fear conditioning. The inter-trial interval was 90 s. One minute after training, mice were returned to their home cages.

Recall of fear conditioning or extinction was performed in a cage with a context different from the cage used in fear conditioning or extinction by changing the covering of the walls and floor of the cage. The cage was equipped with non-shocking grids. No scent was put in the cage. Mice were habituated for 2 min and received five presentations of the same auditory tone that was used in fear conditioning or extinction. The inter-trial interval was 90 s. One minute after training, mice were returned to their home cages.

Reconditioning was performed in a cage with a context different from the cage used in fear conditioning by changing the covering of the walls and floor of the cage. The cage was equipped with shocking grids. Mice were habituated for 2 min on shocking grids and then received seven presentations of 30-s same auditory tone co-terminating with a 2-s footshock as fear conditioning. The intertrial interval was 90 s. One minute after reconditioning, mice were returned to their home cages.

### Inactivation of motor and auditory cortices

1-month-old C57BL/J mice were anaesthetized with ketamine and xylazine. Guide cannulas were implanted bilaterally into the auditory (2.5 mm posterior to Bregma, 4.3 mm lateral to the midline, 500 µm in depth), or primary motor cortex (1.6 mm anterior to Bregma, 1.6 mm lateral to the midline, 500 µm in depth). The guide cannulas were held with dental acrylic cement. The mice were subjected to behavioral training three days after surgery. To examine the involvement of cortical regions in fear extinction generalization, mice were subjected to three different CS-USs on day 0, day 2, and day 4, respectively, fear extinction with fifteen CS2-alone presentations on day 6, and recall test on day 8. Muscimol was infused into the auditory, or motor cortex 1 h before fear extinction. For infusion, an injection cannula of the same length as the guide cannula was inserted into the guide cannula. 500 nl muscimol (1 µg/µl) was infused into the cortex bilaterally in 5 min through the injection cannula connected with a microsyringe driven by a microinfusion pump. To determine the position of cannula, mice were sacrificed after behavioral training and brains were cut into sections at 30 µm. Sections were mounted on slides and stained with toluidine blue. Cannula position was identified using a light microscope and the area of the auditory cortex or motor cortex was determined with the aid of atlas of the mouse brain^82^.

### In vivo imaging and analysis of dendritic spine remodeling

1-month-old YFP-H line mice were used for in vivo imaging of dendritic spine remodeling. The YFP-positive pyramidal neurons in the cortex of YFP-H line mice are largely located in layer 5, and to small extent, in layer 6^83,84^. However, only layer 5 YFP-positive neurons extend their dendrites to the superficial cortical layer^85^. Similar to previous imaging studies of layer 5 pyramidal neurons using YFP H line mice^27,43^, we imaged the apical dendrites located within the first 100 μm of the cortical surface. Therefore, the spine remodeling on apical dendrites observed in this study is limited to layer 5 pyramidal neurons.

Details of the procedures for in vivo imaging and data analysis have been described in the previous studies^27,48^. Briefly, 1-month-old YFP-expressing mice were anaesthetized with ketamine and xylazine. Skull was glued to a stainless steel plate and a small region of the motor cortex (1.5 mm anterior to Bregma, 1.5 mm lateral to the midline, ~200 µm in diameter) was thinned to about 20 µm using a high-speed microdrill. A two-photon microscope (Olympus FV1000MPE) equipped with a Ti:Sapphire laser (MaiTai DeepSee Spectra Physics) tuned to 920 nm was used to acquire images (60X water immersion lens, N.A. = 1.1) of apical dendrites of pyramidal neurons. The thinned region was re-thinned with microsurgical blades for repeated imaging at different intervals. The map of the brain vasculature was used to relocate the imaged region. Image acquisition was performed using FV10-ASW v.3.0 software.

Data analysis was performed with the ImageJ software (Fiji 1.0). Briefly, the same apical dendritic segments were identified from three-dimensional stacks taken from images at different intervals. Three-dimensional stacks were used to ensure that tissue movements and rotation did not influence spine identification. The spine remodeling was identified and analyzed in pairs of images acquired over a certain interval. Filopodia were identified as thin structures (head intensity generally lower than one third of adjacent dendritic shaft intensity, ration of head diameter to neck diameter <1.2:1 and ration of length to neck diameter >3:1). The remaining dendritic protrusions were classified as spines. Spines were considered the same between pairs of images if they were within 0.7 µm of their expected positions. An eliminated spine was a spine that appeared in the image acquired at preceding time point but not the image acquired in the succeeding time point. A newly-formed spine was a spine that appeared in the image acquired in the succeeding time point but not in the image acquired at preceding time point. The rate of spine formation or elimination was measured as the number of spines formed or eliminated divided by the number of existing spines in the image acquired at preceding time point. To examine the distance of newly-formed spines after fear extinction relative to spines eliminated after fear conditioning, pairs of a new spine formed after fear extinction and a proximate spine eliminated after fear conditioning were taken from images and their distance was measured.

To examine the spine remodeling induced by different CS-US pairings on individual neurons, multiple apical dendritic branches from the same neuron were identified from three-dimensional stacks of images. On average, 58 ± 3, 55 ± 3, and 47 ± 4 spines were analyzed per neuron per mouse in CS1-US, CS2-US and CS3-US paired groups respectively (CS1-US: 28 mice; CS-US2: 28 mice; CS3-US: 14 mice). To identify individual neurons with spine elimination induced by CS-US pairing, mean plus two times standard deviation of spine elimination rate of individual neurons in untrained control mice was calculated and set as a threshold. Individual neurons in CS-US paired mice were classified as those with spine elimination induced by CS-US pairing if the spine elimination rate of individual neurons exceeded the set threshold.

To examine spine remodeling on individual dendritic branches of individual neurons, separated apical dendritic branches from the same parent dendrite were identified from three-dimensional stacks of images. Analysis of spine remodeling was done on individual apical branches from the branching point to the distal end of the branch. To determine the individual branches with spine elimination induced by CS-US pairing, mean plus two times standard deviation of spine elimination rate of individual branches in untrained control mice was calculated and set as a threshold. Individual dendritic branches in CS-US paired mice were classified as those with spine elimination induced by CS-US pairing if the spine elimination rate of individual branches exceeded the set threshold.

To estimate the expected proportion of branches with chance occurrence of spine elimination induced by two different CS-US pairings (Supplementary Fig. 8), we first calculated the expected number of branches with chance occurrence of spine elimination induced by two different CS-US pairings as “the number of branches with spine elimination induced by one of two different CS-US pairings × (the number of branches with spine elimination induced by the other CS-US pairing/the number of total branches), assuming that each CS-US pairing induced spine elimination randomly on branches. The expected number of branches with chance occurrence of spine elimination induced by two different CS-US pairings was then divided by the number of branches with spine elimination induced by one of two different CS-US pairings to obtain the expected percentage in Supplementary Fig. 8. In addition, the expected proportion of branches with chance occurrence of spine elimination induced by two different CS-US pairings was also estimated using a bootstrapping procedure, resampling, with replacement, the spine elimination rate on individual dendritic branches after two different CS-US pairings 1000 times. Each time the proportion of branches with spine elimination induced by two different CS-US pairings relative to branches with spine elimination induced by each of different CS-US pairings was calculated.

### In vivo imaging and analysis of somatic Ca^2+^ activity

1-month-old GCaMP6-expressing transgenic mice were used for Ca^2+^ imaging experiments. 24 h before imaging, mice were anaesthetized and a head holder was attached as before^31^. A craniotomy was made above the motor cortex (1.5 mm anterior to Bregma, 1.5 mm lateral to the midline, ~500 µm in diameter). A glass coverslip was placed on the craniotomy and was glued to the skull to reduce the brain motion.

Ca^2+^ imaging was performed in awake, head-restrained mice. Mice were exposed to 2–3 auditory tone presentations (80 dB, 30 s), and a two-photon microscope (Olympus FV1000MPE) equipped with a Ti:Sapphire laser (MaiTai DeepSee Spectra Physics) tuned to 920 nm was used to acquire images (25× water immersion lens, N.A. = 1.05) of somas of pyramidal neurons. The GCaMP6-positive neurons in the cortex of GCaMP6S Line 3 mice are located in layer 2/3, layer 5 and layer 6^80^. We identified the GCaMP6-positive neurons located within 500-600 μm from the pial surface for recording. Neurons located within this depth in the motor cortex are recognized as layer 5 pyramidal neurons^86,87^. The imaging period was divided into a 30-s pre-CS period and a 30-s CS presentation period.

The fluorescence signal of each soma was measured by averaging all pixels within the circular ROIs covering the soma. The baseline (*F*_0_) of the fluorescence trace was estimated by detecting inactive portions of the trace using an iterative procedure described before^88,89^. Briefly, we smoothed (loess) the raw fluorescence trace and subtracted the smooth trace from the raw trace, denoted as preliminary *F*_0_. Two times the standard deviation of the preliminary *F*_0_ trace was set as a threshold for detecting inactive portions in the raw fluorescence trace. The inactive portions were concatenated and the procedure was repeated once again. The resulting inactive portions in the raw fluorescence trace were then used to yield the *F*_0_. The Δ*F*/*F*_0_ was calculated as Δ*F*/*F*_0_ = (*F* − *F*_0_)/*F*_0_.

To determine the tone response of neurons, the Δ*F*/*F*_0_ of each soma during pre-CS and CS presentation periods larger by three times the standard deviation of inactive portions of raw fluorescence trace yielding *F*_0_ during the pre-CS presentation period was selected. The selected values during the pre-CS or CS presentation period were summed respectively to represent the total soma activity. Neurons were classified as those with increased responses to CS if the summed Δ*F*/*F*_0_ during CS presentation period was two times higher than that during pre-CS presentation period. Neurons were classified with those with decreased response to CS if the summed Δ*F*/*F*_0_ during CS presentation period was two times lower than that during pre-CS presentation period.

### In vivo imaging and analysis of spine Ca^2+^ activity

To examine spine Ca^2+^ activity, AAV2-Syn-Cre, Cre-dependent AAV2/1-CAG-Flex-GCaMP6S and Cre-dependent AAV2/1-CAG-Flex-tdTomato (University of Pennsylvania Gene Therapy Program Vector Core) were used to drive GCaMP6 and tdTomato co-expression in cortical pyramidal neurons. AAV2-Syn-Cre virus was diluted in artificial cerebrospinal fluid (ACSF) (0.1%) and was then mixed with the other two viruses so that the final solution was composed of ~80% GCaMP6S, ~17% tdTomato and ~13% diluted Cre. This viral combination enabled sparse double labeling of pyramidal neurons within the imaging field. 0.2 µl of mixture of viruses were injected into the brains of neonatal mice during the first 12–24 h after birth.

One month after virus expression, mice with GCaMP6 and tdTomato co-expression in the motor cortex were identified and used for imaging experiments. 24 h before imaging, mice were anaesthetized and a head holder was attached. After dendritic and spine imaging, we tracked the apical dendrites down to the somas of GCaMP6 and tdTomato co-labeled neurons to ensure that imaged neuronal somas were located within a depth of 500–600 μm from the pial surface in the motor cortex.

Imaging was performed in awake, head-restrained mice. A small region of the motor cortex was thinned for imaging. Mice were exposed to 2–3 auditory tone presentations (1 kHz, 80 dB, 15 s) and a two-photon microscope (Bruker) equipped with a Ti:Sapphire laser (MaiTai DeepSee Spectra Physics) tuned to 920 nm was used to acquire images (25× water immersion lens, N.A. = 1.05) of the apical dendrites pyramidal neurons. The fluorescence of GCaMP6 and tdTomato co-expressing in dendritic spines were separated into green and red channels. The imaging period was divided into a 15-s pre-CS and a 15-s CS presentation period. Three-dimensional stacks were taken to identify the same dendritic segments for repeated imaging at different intervals and investigate spine remodeling on these dendritic segments. Image acquisition was performed using Prairie View 5.4 software. The Ca^2+^ fluorescence signal of each spine was measured by averaging all pixels within the circular ROIs covering the spine. The Δ*F*/*F*_0_ of each spine during pre-CS or CS presentation periods larger by three times the standard deviation of inactive portions of raw fluorescence trace yielding *F*_0_ during the pre-CS presentation period was selected. The selected values during the pre-CS or CS presentation period were summed respectively to represent total spine activity. Spines were classified as those with increased responses to CS if the summed values during CS presentation period was higher than that during pre-CS presentation period. Spines were classified as those with decreased response to CS if the summed values during CS presentation period was lower than that during pre-CS presentation period. Spines were classified as no activity if spine Δ*F*/*F*_0_ over the imaging period was within three times the standard deviation of inactive portions of raw fluorescence trace yielding *F*_0_.

### Manipulating activity of SST INs

Cre-dependent AAV2-hSyn-DIO-hM3D(Gq)-mCherry or Cre-dependent AAV2-hSyn-DIO-hM4D(Gi)-mCherry (University of North Carolina Vector Core) was used to drive DREADD expression in SST Cre mice crossed with YFP H line mice. Viruses were injected into layer 5 of the motor cortex (1.6 mm anterior to Bregma, 1.6 mm lateral to the midline, ~500 µm in depth) of 3-week-old mice. Two weeks after virus expression, mice were used for behavioral and imaging experiments. CNO (Sigma Aldrich) was dissolved in saline to a concentration of 0.5 mg ml^−1^, and administered by intraperitoneal injection to each mouse (0.3 ml per 30 g body weight) 30 min before behavioral or imaging experiments.

To validate the efficacy of SST INs manipulation with DREADD-hM3D(Gq) or DREADD-hM4D(Gi), two viruses (AAV2/1-Flex-GCaMP6s and AAV2-hSyn-DIO-hM3D(Gq)-mCherry; or AAV2/1-Flex-GCaMP6s and AAV2-hSyn-hM4D(Gi)-mCherry) were mixed and injected into the motor cortex of SST Cre mice 2 weeks before imaging. Layer 5 SST INs (~500–600 µm below the cortical surface) were imaged for 1 min under the quiet resting state. CNO was then administered to mice, and the same neurons were imaged for 1 min under the quiet resting state again 30 min after CNO administration. The Ca^2+^ fluorescence signal during 1-min quiet period was averaged to compare the somatic activity before CNO or after CNO.

To detect the specificity of SST IN manipulation in the motor cortex, AAV2-hSyn-DIO-hM3D(Gq)-mCherry was injected into the motor cortex of SST Cre mice. Two weeks later after AAV expression, mice were anaesthetized and perfused in 4% paraformaldehyde in PBS. The brain was removed and post-fixed for 1 h in the same fixative at 4 °C. Coronal sections (150 µm thick) were cut on a vibratome. Sections were then blocked with for 1 h with 10% normal goat serum and incubated overnight with primary antibodies against RFP (1:750, Rockland Immunochemicals). After wash with PBS/0.05% Tween-20, the sections were incubated with Alexa Fluor-conjugated goat anti-rabbit IgG secondary antibodies (1:500, Life Technologies) in PBS for 2 h. Sections were examined on a confocal microscope (Zeiss LSM 700). Number of SST-mcherry positive somas was counted in the slice in the 1 mm^2^ area on the focal plane with the largest immunoreactivity.

### Statistics

Two-tailed Student’s *t* test was used to test for differences between groups when their distributions passed tests for normality (D’Agostino-Pearson normality test and Anderson-Darling test). Mann–Whitney *U* test and Wilcoxon matched-pairs signed rank test were used to test for differences between groups when their distributions did not pass tests for normality. Chi-Square test was used to compare difference in the proportion of spines or neurons. Significant levels were set at *P* < 0.05. Statistical analyses were performed using GraphPad Prism 8 and Microsoft Excel 16.36.

### Reporting summary

Further information on research design is available in the Nature Portfolio Reporting Summary linked to this article.

## Supplementary information

Supplementary Information

Reporting Summary

## Data Availability

We declare that all data supporting the findings of this study are provided within the paper and its supplementary information. Underlying data of all figures are provided in the Source Data file with this paper and data are fully available from the corresponding author on request. A source data file is provided with this paper. Source data are provided with this paper.

## Source data

Source Data
