# Supplementary material for: Generalized extinction of fear memory depends on co-allocation of synaptic plasticity in dendrites
Source: Nat Commun. 2023 Jan 31;14:503. doi: 10.1038/s41467-023-35805-9 (PMC9889816; doi:10.1038/s41467-023-35805-9)
Supplement: Supplementary file 1 — Supplementary Information [file 41467_2023_35805_MOESM1_ESM.pdf]

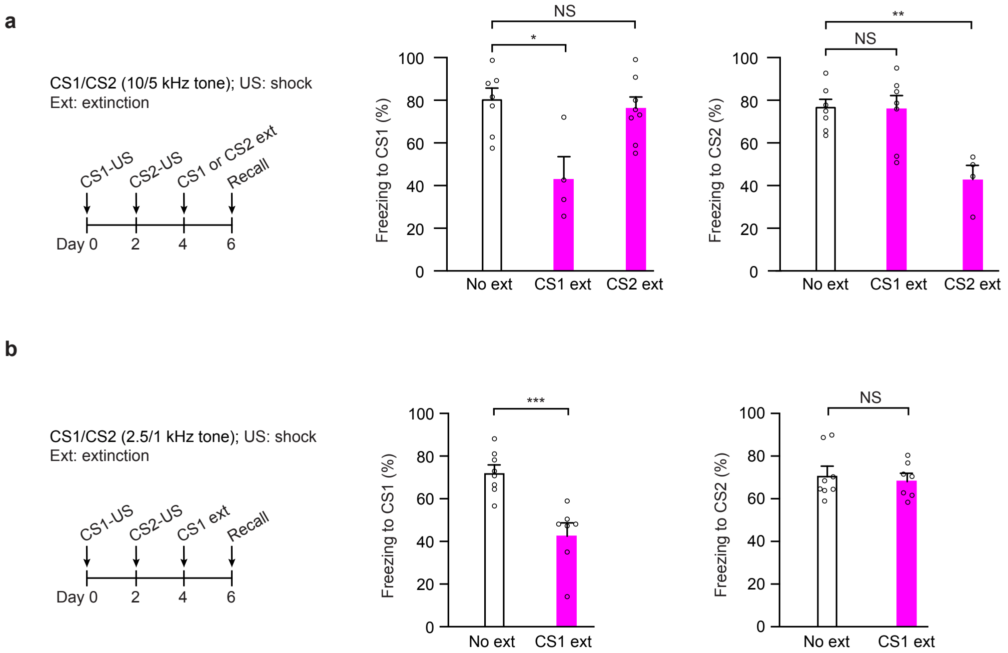

Supplementary Fig. 1 In mice subjected to CS1 and CS2 paired with US, fear extinction with one CS does not change the freezing response to the other CS. a. Mice (YFP-H line) were subjected to CS1 (10 kHz)-US and CS2 (5 kHz)-US pairings followed by CS1 or CS2 extinction. The freezing response to CS1 after CS2 extinction was comparable to that after no extinction (CS1: CS1 or CS2 extinction vs. no extinction, P = 0.0242 or P = 0.6126, n = 7, 4 and 8 mice in no extinction, CS1 extinction and CS2 extinction groups respectively; Mann–Whitney U test). No significant difference in the freezing response to CS2 between CS1 extinction and no extinction groups (CS2: CS1 or CS2 extinction vs. no extinction, P = 0.8048 or P = 0.0061; n = 7, 7 and 4 mice in no extinction, CS1 extinction and CS2 extinction groups respectively; Mann–Whitney U test). **b.** Mice (YFP-H line) were subjected to CS1 (2.5 kHz)-US and CS2 (1 kHz)-US pairings followed by CS1 extinction. The freezing response to CS2 after CS1 extinction was comparable to that after no extinction (CS1: P = 0.0006; CS2: P = 0.8665; Mann–Whitney U test; n = 8 and 7 mice in no extinction and CS1 extinction groups respectively). Error bars, ± S.E.M. All statistical tests were performed two-sided. NS: not significant. * $P < 0.05$; ** $P < 0.01$; *** $P < 0.001$.

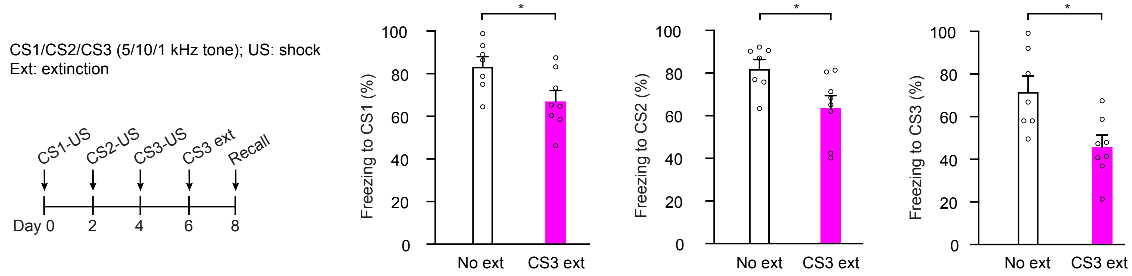

Supplementary Fig. 2 In mice subjected to CS1 (5 kHz), CS2 (10 kHz) and CS3 (1 kHz) paired with US, fear extinction with CS3 reduces the freezing response to other CSs. Mice (YFP-H line) were subjected to CS1-US, CS2-US and CS3-US pairings followed by CS3 extinction. Fear extinction with CS3 significantly reduced the freezing response to CS1 and CS2 (CS1: $P = 0.0401$; CS2: $P = 0.0289$; CS3: $P = 0.014$; Mann–Whitney U test; n = 7 and 8 mice in no extinction and CS3 extinction groups respectively). Error bars, ± S.E.M. Statistical tests were performed two-sided. * $P < 0.05$.

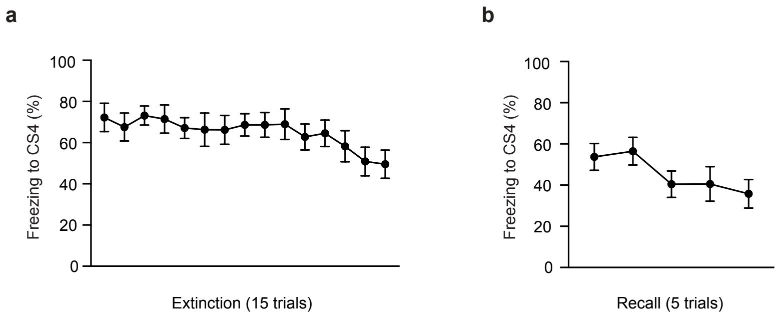

Supplementary Fig. 3 Freezing response during the CS4 extinction period and during the recall after CS4 extinction. a. Freezing response during the CS4 extinction period in mice (YFP-H line) receiving CS1-US, CS2-US, CS3-US and CS4-US pairings (CS1: 1 kHz; CS2: 10 kHz; CS3: 5 kHz; CS4: 2.5 kHz; n = 11 mice). **b.** Freezing response during the recall test two days after CS4 extinction (n = 11 mice). Error bars, ± S.E.M.

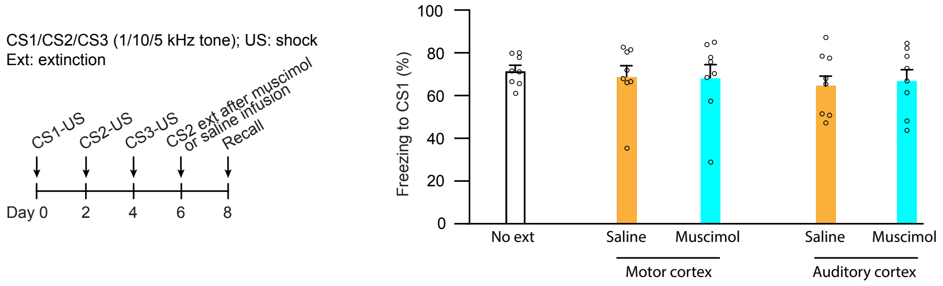

Supplementary Fig. 4 In mice subjected to three different CS-US pairings, infusion of muscimol into auditory or motor cortex before CS2 extinction does not change the freezing response to CS1 after CS2 extinction. Mice (YFP-H line) were subjected to three different CS-US pairings followed by CS2 extinction (CS1: 1 kHz; CS2: 10 kHz; CS3: 5 kHz). Muscimol or vehicle was infused bilaterally into the auditory or motor cortex before CS2 extinction. The freezing response to CS1 after CS2 extinction in mice with muscimol infusion into the motor cortex or auditory cortex was comparable to that in vehicle treated group or that in no extinction group (motor cortex: $P = 0.7984$ and 0.9814 respectively; auditory cortex: $P = 0.9591$ respectively; Mann–Whitney U test; n = 8 mice in each group). Error bars, ± S.E.M. Statistical tests were performed two-sided.

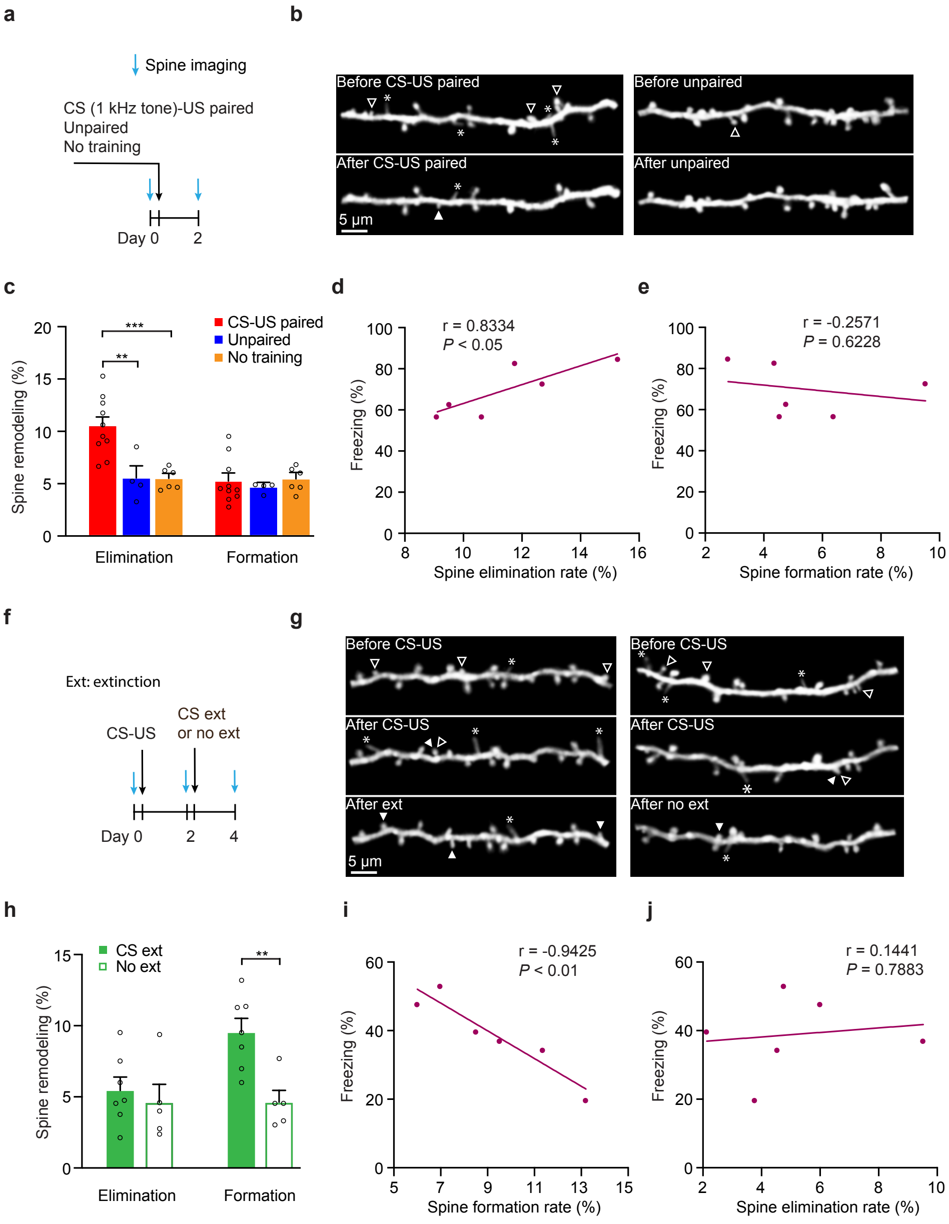

a
Spine imaging
CS (1 kHz tone)-US paired
Unpaired
No training
Day 0
2
b
Before CS-US paired
After CS-US paired
Before unpaired
After unpaired
5 µm
c
CS-US paired
Unpaired
No training
Spine remodeling (%)
Elimination
Formation
d
r = 0.8334
P < 0.05
Freezing (%)
Spine elimination rate (%)
e
r = -0.2571
P = 0.6228
Freezing (%)
Spine formation rate (%)
f
Ext: extinction
CS-US
CS ext
or no ext
Day 0
2
4
g
Before CS-US
After CS-US
After ext
After no ext
5 µm
h
CS ext
No ext
Spine remodeling (%)
Elimination
Formation
i
r = -0.9425
P < 0.01
Freezing (%)
Spine formation rate (%)
j
r = 0.1441
P = 0.7883
Freezing (%)
Spine elimination rate (%)

Supplementary Fig. 5 Fear conditioning and extinction induce spine elimination and formation on apical dendrites of layer 5 pyramidal neurons in the motor cortex, respectively. **a.** Experimental design to examine spine remodeling in YFP-H line mice subjected to CS (1 kHz) paired with US, unpaired stimuli or no training. **b.** Representative images of spine remodeling induced by CS-US pairing or unpaired stimuli on apical dendrites of layer 5 pyramidal neurons. The hollow triangles indicate spines eliminated in the succeeding image. The solid triangles indicate newly-formed spines when compared to preceding image. The asterisks indicate filopodia. Experiments were repeated in10 and 4 mice in paired and unpaired group respectively with similar results. **c.** The rate of dendritic spine elimination, but not formation, was significantly higher in CS-US paired group than that in unpaired stimuli or no training group (P = 0.008 or P = 0.0005 respectively, Mann–Whitney U test; n = 10, 4 and 6 mice from paired, unpaired and no training groups). **d-e.** The rate of spine elimination (**d**), but not formation (**e**), after CS-US pairing was positively correlated with the freezing responses to CS (P = 0.0389, Pearson's correlation). **f.** Experimental design to examine spine remodeling in mice subjected to CS-US pairing and CS extinction or no extinction. **g.** Representative images of spine remodeling induced by CS-US pairing and CS extinction or no extinction on apical dendrites of layer 5 pyramidal neurons. The hollow triangles indicate spines eliminated in the succeeding image. Experiments were repeated in 7 and 5 mice in extinction and no extinction groups respectively with similar results. The solid triangles indicate newly-formed spines when compared to preceding image. The asterisks indicate filopodia. **h.** The rate of spine formation, but not elimination, was significantly higher in the extinction group as compared to that in no extinction group (P = 0.0088, Mann–Whitney U test; n = 7 and 5 mice from CS extinction and no extinction groups respectively). **i-j** The rate of spine formation (**i**), but not elimination (**j**), after extinction was inversely correlated with the freezing response to CS (P = 0.0048, Pearson's correlation). Error bars, ± S.E.M. All statistical tests were performed two-sided. ** $P < 0.01$; *** $P < 0.001$.

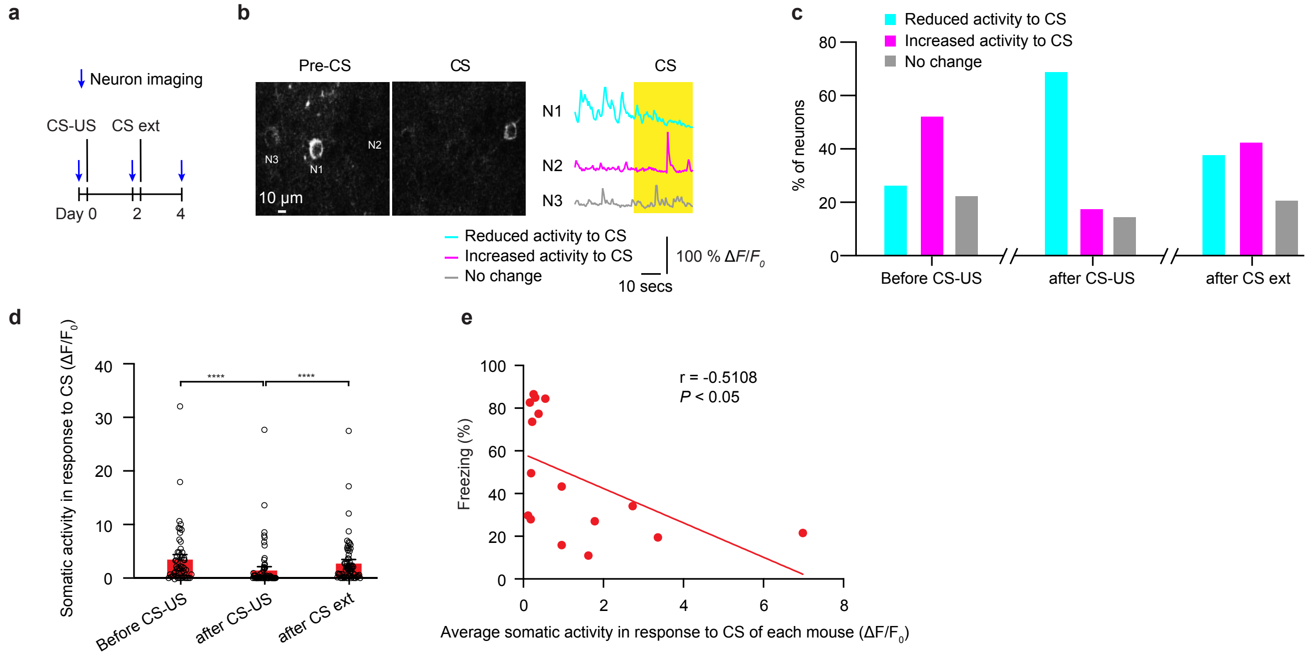

Supplementary Fig. 6 Fear conditioning reduces while fear extinction increases somatic Ca^{2+} activity of layer 5 pyramidal neurons in the motor cortex. a. Experimental design to examine somatic Ca^{2+} activity of layer 5 pyramidal neurons before and during CS presentation in mice subjected to CS (1 kHz)-US pairing followed by CS extinction. **b.** Representative images of somatic Ca^{2+} fluorescence before and during CS presentation in GCaMP6S line 3 mice. Somatic Ca^{2+} changes were measured by $\Delta F/F_0$. Experiments were repeated independently in 64 neurons in 4 mice with similar results. **c.** Percentage of neurons with reduced or increased activity before fear conditioning, after fear conditioning and after fear extinction. After CS-US pairing, a larger percentage of somas exhibited reduced somatic Ca^{2+} activity in response to CS when compared to that before fear conditioning (reduced activity: $P < 0.0001$; increased activity: $P < 0.0001$; chi-square test; n = 64 and 54 neurons from 4 mice respectively). After fear extinction, a smaller percentage of somas showed reduced activity to CS when compared to that after CS-US pairing (reduced activity: $P = 0.0004$; increased activity: $P = 0.002$; chi-square test; n = 64 neurons from 4 mice respectively). **d.** Changes in somatic Ca^{2+} activity during the CS presentation. Somatic Ca^{2+} activity in response to CS was significantly lower after fear conditioning when compared to that before fear conditioning but significantly higher after fear extinction ($P < 0.0001$ respectively, Mann–Whitney U test; n = 54 neurons before fear conditioning, 64 neurons after fear conditioning and 64 neurons after fear extinction respectively). **e.** Inverse correlation between the somatic Ca^{2+} activity in response to CS and the behavioral response in mice after fear conditioning or extinction. $\Delta F/F_0$ during the CS presentation period of each analyzed neuron was averaged in each mouse ($P = 0.0432$, Pearson's correlation). Error bars, ± S.E.M. All statistical tests were performed two-sided. **** $P < 0.0001$.

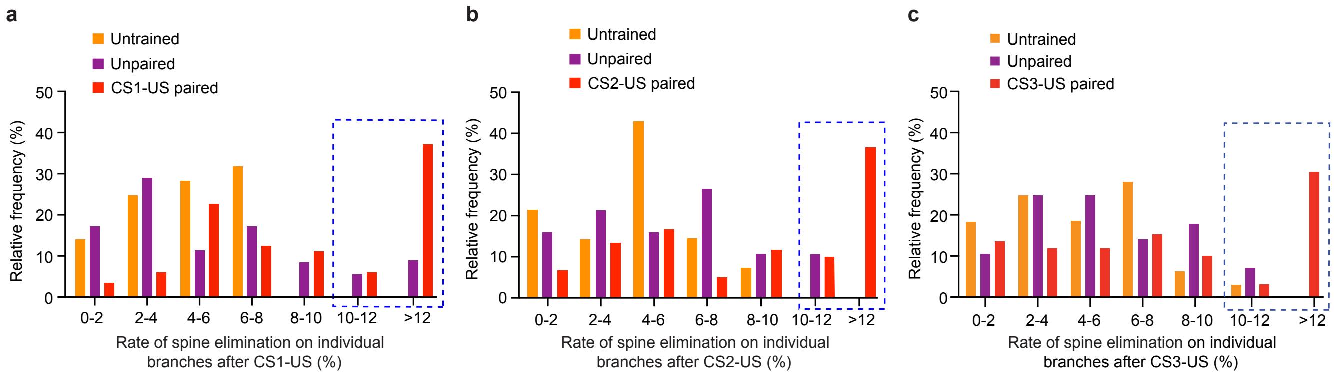

Supplementary Fig. 7 Fear conditioning induces spine elimination on a subset of dendritic branches. (a-c) Distribution of spine elimination rate induced by CS1-US pairing (**a**), by CS2-US pairing (**b**) or by CS3-US pairing (**c**) on individual dendritic branches (CS1: 1kHz; CS2: 10 kHz; CS3: 5 kHz) in YFP-H line mice. Only a fraction of individual branches in CS-US paired mice showed the spine elimination rate higher than the mean plus 2 times standard deviation of that in untrained control mice. Blue frames indicate a subset of branches in fear conditioned mice with the spine elimination rate higher than the mean plus 2 times standard deviation of that in untrained control mice (total 78, 60 and 58 branches in CS1-US, CS2-US and CS3-US paired mice respectively; total 28, 14 and 32 branches in untrained control mice respectively).

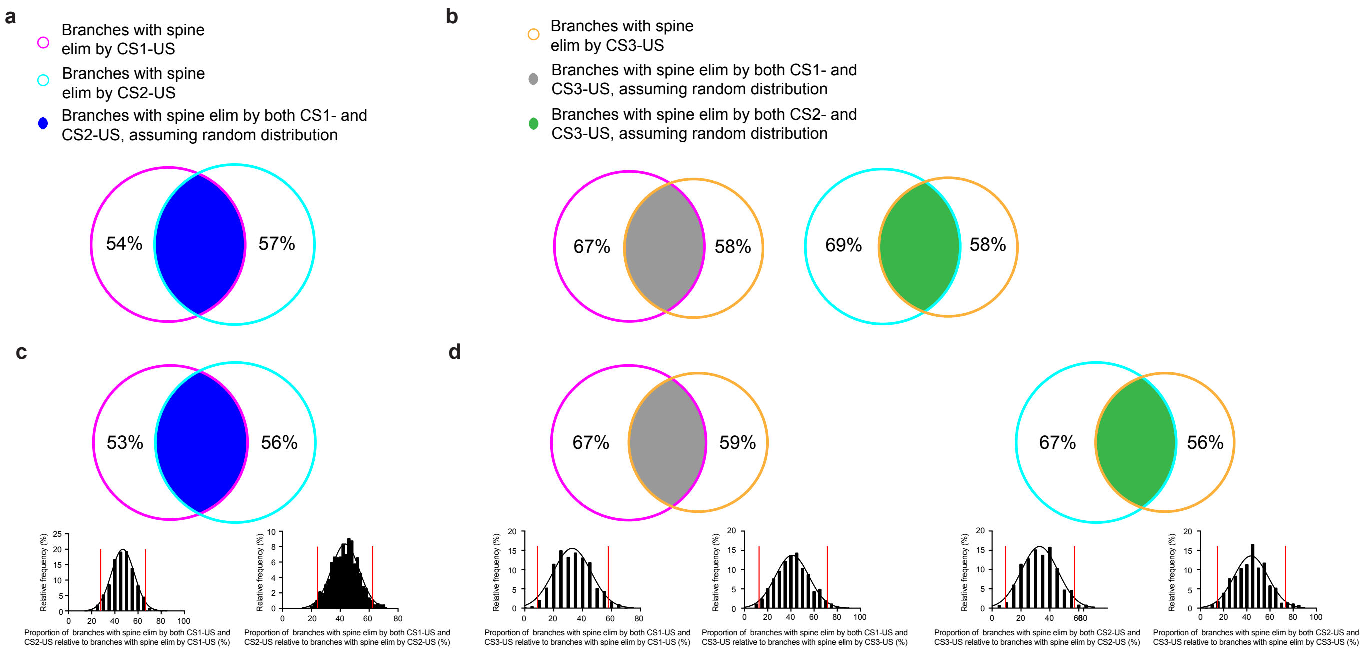

Supplementary Fig. 8 The chance occurrence of spine elimination induced by different CS-US pairings on the same dendritic branches. a. When YFP-H line mice were subjected to two different CS-US pairings (CS1: 1kHz; CS2: 10 kHz), assuming that CS2-US induces spine elimination randomly on branches with or without spine elimination induced by CS1-US pairing, we would expect that ~46% of branches with spine elimination induced by CS1-US would show spine elimination by CS2-US. **b.** When YFP-H line mice were subjected to three different CS-US pairing (CS1: 1kHz; CS2: 10 kHz; CS3: 5 kHz), assuming that CS3-US pairing induces spine elimination randomly on branches with or without spine elimination induced by CS1-US or CS2-US pairing, we would expect that ~33% of dendritic branches with spine elimination by CS1-US pairing or 31% of branches with spine elimination by CS2-US pairing would show spine elimination by CS3-US pairing. **c.** The spine elimination rate on individual dendritic branches after CS1-US and CS2-US pairings was resampled using bootstrapping procedure 1000 times. Each time the proportion of branches with spine elimination induced by both CS1-US and CS2-US pairings relative to branches with spine elimination induced by CS1-US pairing or by CS2-US pairing was calculated. The up panel indicates the average proportion of branches with spine elimination induced by both CS1-US and CS2-US pairings over 1000 resamples. The down panel indicates the distribution of the proportion of branches with spine elimination induced by both CS1-US and CS2-US pairings for 1000 resamples. The red lines indicate 95% confidence interval for the population of 1000 resamples. **d.** The spine elimination rate on individual dendritic branches after CS1-US, CS2-US and CS3-US pairings was resampled using bootstrapping procedure 1000 times. Each time the proportion of branches with spine elimination induced by both CS1-US and CS3-US pairings relative to branches with spine elimination induced by CS1-US pairing or by CS3-US pairing was calculated. The proportion of branches with spine elimination induced by both CS2-US and CS3-US pairings relative to branches with spine elimination induced by CS2-US pairing or by CS3-US pairing was also calculated.

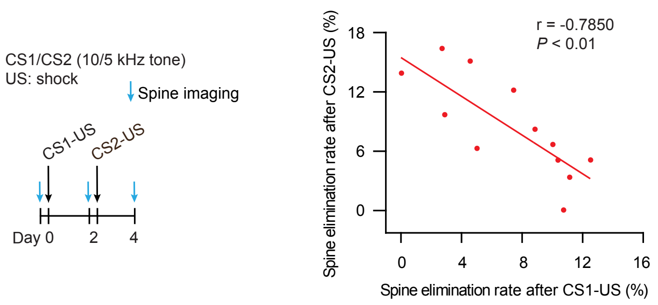

Supplementary Fig. 9 In mice subjected to CS1 (10 kHz) and CS2 (5 kHz) paired with US, the rate of spine elimination induced by two different CS-USs is inversely correlated at the dendritic branch level. YFP-H line mice were fear conditioned with CS1-US and CS2-US pairings (CS1: 10 kHz; CS2: 5 kHz). The rate of spine elimination after CS2-US pairing on individual branches was inversely correlated with that after CS1-US pairing ($P = 0.0025$, Pearson's correlation; n = 12 branches). Statistical test was performed two-sided.

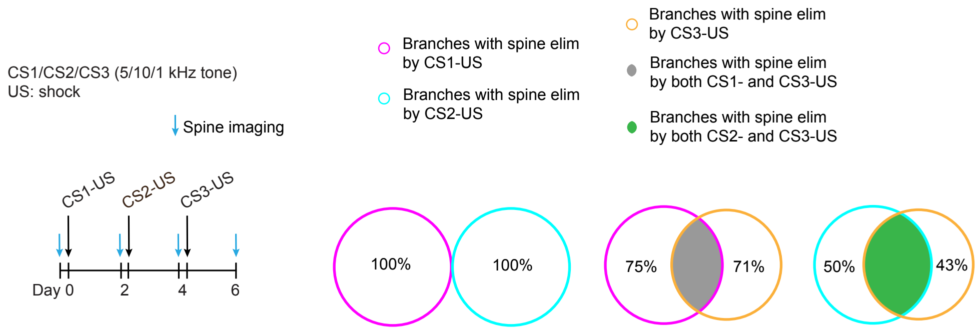

Supplementary Fig. 10 In mice subjected to CS1 (5 kHz), CS2 (10 kHz) and CS3 (1 kHz) paired with US, a fraction of dendritic branches undergo spine elimination induced by both CS2-US and CS3-US pairings. YFP-H line mice were first fear conditioned with CS1 (5 kHz)-US and CS2 (10 kHz)-US pairings and then subjected to CS3 (1 kHz)-US pairing. ~25% and ~50% of dendritic branches with spine elimination induced by CS1-US pairing or by CS2-US pairing showed spine elimination induced by CS3-US pairing (total 20 branches after three different CS-USs).

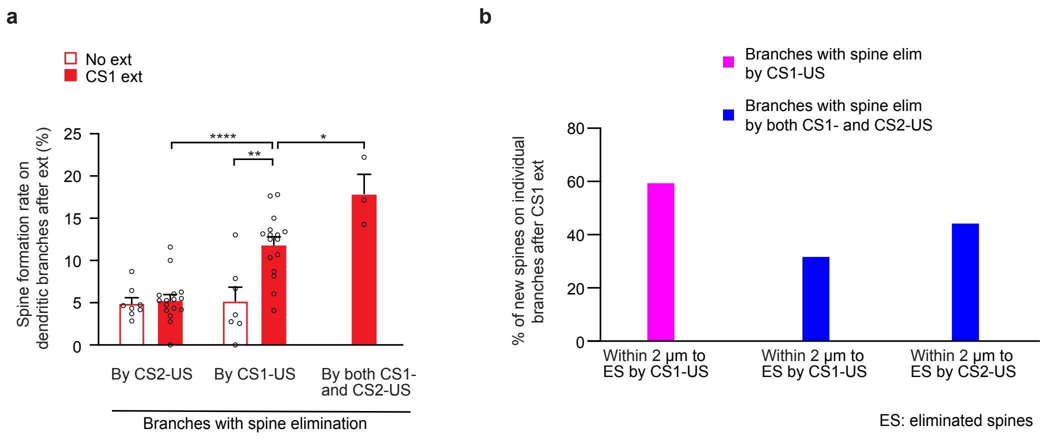

Supplementary Fig. 11 In mice subjected to two different CS-US pairings followed by CS1 extinction, CS1 extinction induces spine formation on branches with spine elimination induced by only CS1-US pairing and branches with spine elimination induced by both CS1-US and CS2-US pairings. a. Comparisons of spine formation rates after CS1 extinction on branches with spine elimination induced by two different CS-US pairings (CS1: 1 kHz, CS2: 10 kHz; spine formation rate after CS1 extinction on branches with spine elimination induced by only CS1-US pairing vs. by only CS2-US pairing or by both CS1-US and CS2-US pairings, $P < 0.0001$ and $P = 0.032$ respectively; spine formation rate after CS1 extinction vs. no extinction on branches with spine elimination induced by only CS1-US pairing, $P = 0.0025$; Mann–Whitney U test respectively; n = 16, 16 and 3 branches with spine elimination induced by only CS1-US pairing or by only CS2-US pairing or by both CS1-US and CS2-US pairings in CS1 extinction group respectively; n = 7 and 8 branches with spine elimination induced by only CS1-US pairing or by only CS2-US pairing in no extinction group respectively). **b.** On branches with spine elimination induced by only CS1-US pairing, the majority of new spines formed after CS1 extinction were located within 2 μm to spines eliminated by CS1-US pairing (n = 51 newly-formed spines from 7 mice). On branches with spine elimination induced by both CS1-US and CS2-US pairings, a large percentage of new spines formed after CS1 extinction were also located within 2 μm to spines eliminated by CS2-US pairing (n = 16 newly-formed spines from 7 mice). Error bars, ± S.E.M. Statistical tests were performed two-sided. * $P < 0.05$; ** $P < 0.01$; **** $P < 0.0001$.

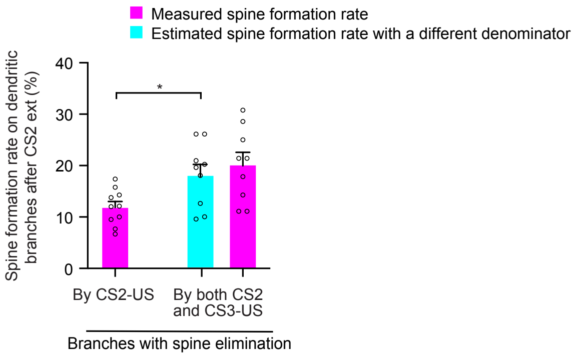

Supplementary Fig. 12 The high spine formation rate after CS2 extinction on the branches with spine elimination induced by both CS2-US and CS3-US pairings is not due to the change in the total number of spines on those branches. To determine whether the high spine formation rate after CS2 extinction on branches with spine elimination induced by both CS2-US and CS3-US pairing is due to change in the total of number of spines (CS1: 1 kHz; CS2: 10 kHz; CS3: 5 kHz), we calculated the spine formation rate as the number of new spines formed after CS2 extinction on branches with spine elimination induced by both CS2-US and CS3-US pairing divided by the number of spines existed before, instead of after, CS3-US pairing. The estimated spine formation rate was significantly higher, when compared to that on branches with spine elimination induced by only CS2-US pairing, suggesting that the high spine formation rate after CS2 extinction on the branches with spine elimination induced by both CS2-US and CS3-US pairings is not due to that more spines are eliminated on those branches (P = 0.0264, unpaired t-test). Error bars, ± S.E.M. Statistical test was performed two-sided. * $P < 0.05$.

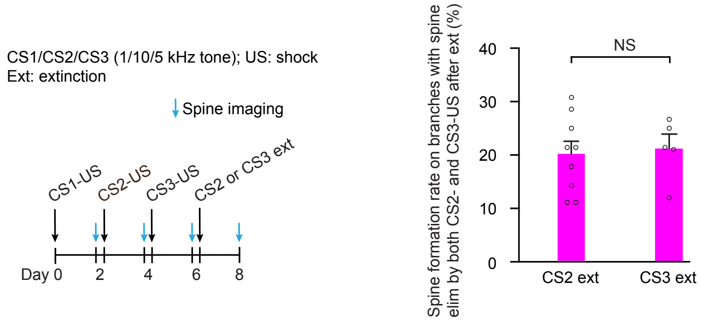

Supplementary Fig. 13 In mice subjected to three different CS-US pairings, on branches with spine elimination induced by both CS2-US and CS3-US pairings, the spine formation rate after CS2 extinction is comparable to that after CS3 extinction. YFP-H line mice were fear conditioned with three different CS-US pairings followed by CS2 or CS3 extinction (CS1: 1 kHz; CS2: 10 kHz; CS3: 5 kHz). On branches with spine elimination induced by both CS2-US and CS3-US pairings, the spine formation rate was comparable after CS2 extinction or CS3 extinction (P = 0.9211, Mann–Whitney U test; n = 9 and 5 branches for CS2 and CS3 extinction groups respectively). Error bars, ± S.E.M. Statistical test was performed two-sided. NS: not significant.

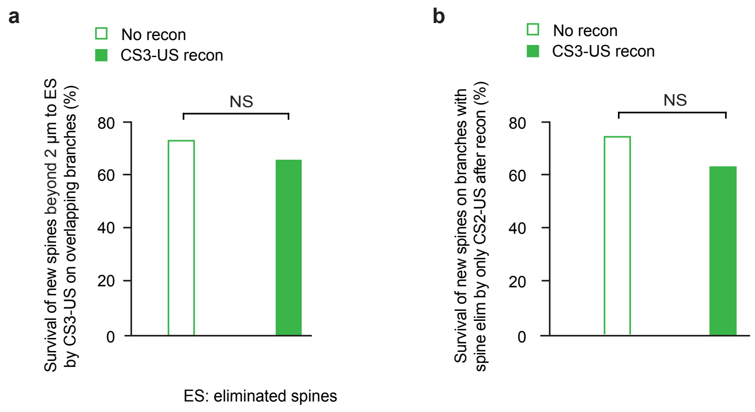

Supplementary Fig. 14 New spines that are formed after CS2 extinction and not located close to the site of spines eliminated after CS3-US pairing were not preferentially eliminated by CS3-US reconditioning. a. On branches with spine elimination induced by both CS2-US pairing and CS3-US pairings (CS1: 1 kHz; CS2: 10 kHz; CS3: 5 kHz), CS3-US reconditioning had no effects on the survival rate of new spines that were formed after CS2 extinction and located beyond 2 μm to spines eliminated after CS3-US pairing ($P = 0.5418$, compared to no reconditioning group, chi square test; n = 32 newly-formed spines from 8 mice in reconditioning group and 26 newly-formed spines from 4 mice in no reconditioning group respectively). **b.** On branches with spine elimination induced by only CS2-US pairing, CS3-US reconditioning had no effects on the survival rate of new spines that were formed after CS2 extinction ($P = 0.3066$, compared to no reconditioning group, chi square test; n = 38 and 35 newly-formed spines in 4 mice respectively). All statistical tests were performed two-sided. NS: not significant.

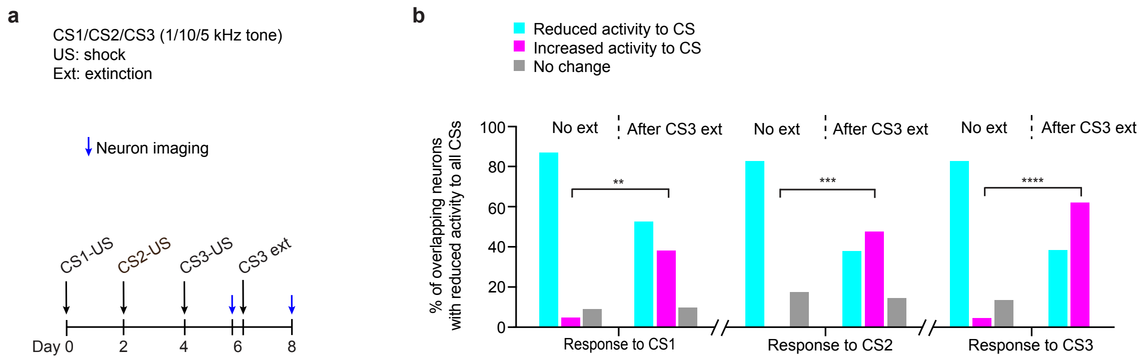

Supplementary Fig. 15 In mice subjected to three different CS-US pairings, the overlapping neurons with reduced activity to all CSs showed increased activity to CS1, CS2 and CS3 during the recall test after CS3 extinction. a. GCaMP6S line 3 mice were fear conditioned with three different CS-US pairings (CS1: 1 kHz; CS2: 10 kHz; CS3: 5 kHz). The overlapping neurons with reduced activity to all CSs were identified and mice were subjected further to CS3 extinction. **b.** After CS3 extinction, neurons with reduced activity to all CSs showed increased somatic activity to all CSs during the recall test, when compared to no extinction controls (CS1, $P < 0.0056$; CS2, $P = 0.0002$; CS3, $P < 0.0001$; chi-square test; n = 21 and 23 neurons in extinction and no extinction groups respectively). All statistical tests were performed two-sided. ** $P < 0.01$; *** $P < 0.001$; **** $P < 0.0001$.

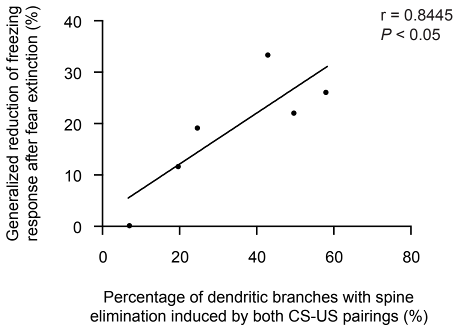

Supplementary Fig. 16 Correlation between the percentage of dendritic branches with spine elimination induced by both CS-US pairings and the generalized reduction of freezing response after fear extinction. In mice subjected to different CS-US pairings followed by CS2 or CS3 extinction (CS1: 1 kHz; CS2: 10 kHz; CS3: 5 kHz or CS1: 5 kHz; CS2: 10 kHz; CS3: 1 kHz), we calculated the generalized reduction of freezing response after fear extinction with CS2 or CS3 as (1-freezing response to a different CS after fear extinction/freezing response to a different CS without fear extinction) x 100%. We also calculated the percentage of dendritic branches with spine elimination induced by CS1-US/CS2-US, CS1-US/CS3-US, or CS2-US/CS3-US. The percentage of dendritic branches with spine elimination induced by CS1-US/CS2-US, CS1-US/CS3-US, and CS2-US/CS3-US was positively correlated with the generalized reduction of freezing response after CS2 or CS3 extinction ($P = 0.0344$, Pearson's correlation). Statistical test was performed two-sided.

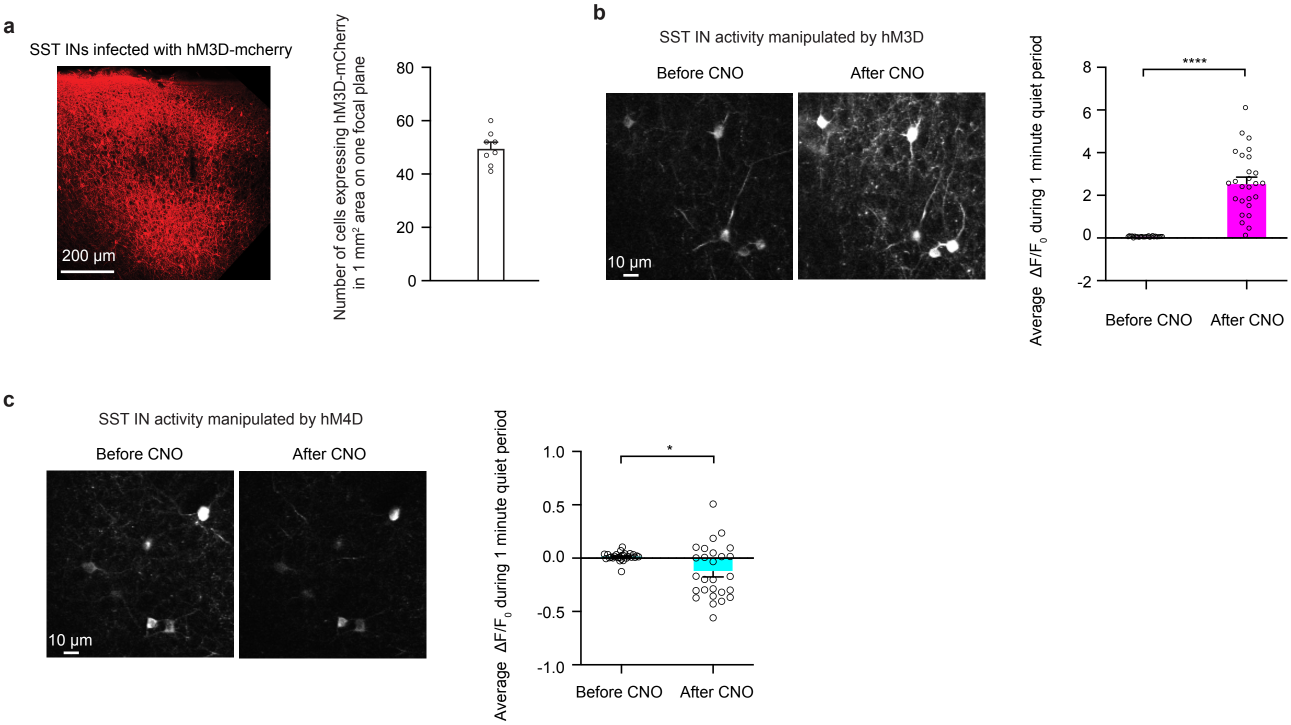

Supplementary Fig. 17 Manipulating the activity of SST INs in the motor cortex with the DREADD/CNO approach. a. Left panel: Images of SST INs infected with hM3D(Gq)-mCherry in the motor cortex. Right panel: Average number of SST INs infected with hM3D(Gq)-mCherry in the 1 mm^2 area on the focal plane with the largest immunoreactivity (n = 8 mice). **b.** Left panel: Representative images of Ca^{2+} activity of SST INs before and after CNO injection to activate hM3D(Gq). Right panel: CNO injection increased the somatic Ca^{2+} activity of SST INs during 1 minute quiet period as compared to that before CNO injection ($P < 0.0001$, Wilcoxon matched-pairs signed rank test; n = 26 neurons in 3 mice). Somatic Ca^{2+} activity was measured as average $\Delta F/F_0$ during the quiet period. **c.** Left panel: Representative images of Ca^{2+} activity of SST INs before and after CNO injection to activate hM4D(Gi). Right panel: CNO injection decreased somatic Ca^{2+} activity of SST INs during 1 minute quiet period when compared to that before CNO injection ($P < 0.0174$, Wilcoxon matched-pairs signed rank test; n = 27 neurons in 3 mice). Error bars, ± S.E.M. All statistical tests were performed two-sided. * $P < 0.05$; **** $P < 0.0001$.

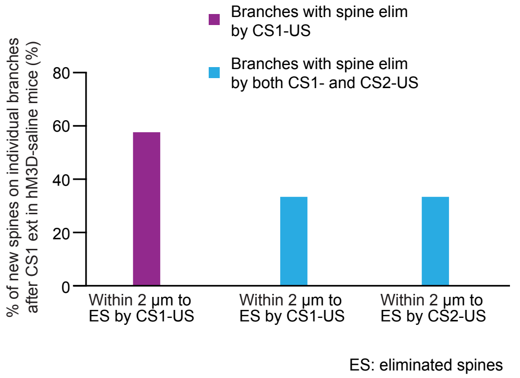

Supplementary Fig. 18 Spatial location of newly-formed spines on individual dendritic branches after CS1 extinction in hM3D(Gq)-saline mice. In hM3D(Gq)-saline mice, ~57% of new spines formed after CS1 extinction on branches with spine elimination induced by only CS1-US pairing were located within 2 μm to spines eliminated by CS1-US pairing (CS1: 1 kHz, CS2: 10 kHz; n = 28 newly-formed spines from 5 mice). On branches with spine elimination induced by both CS1-US and CS2-US pairings, ~33% of new spines formed after CS1 extinction were located within 2 μm to spines eliminated by CS1-US pairing or by CS2-US pairing (n = 9 newly-formed spines from 5 mice).
