# Supplementary material for: Generalized extinction of fear memory depends on co-allocation of synaptic plasticity in dendrites
Source: Nat Commun. 2023 Jan 31;14:503. doi: 10.1038/s41467-023-35805-9 (PMC9889816; doi:10.1038/s41467-023-35805-9)
Supplement: Supplementary file 2 — Reporting Summary [file 41467_2023_35805_MOESM2_ESM.pdf]

nature portfolio

Corresponding author(s): Wenbiao Gan

Last updated by author(s): Dec 15, 2022

Reporting Summary

Nature Portfolio wishes to improve the reproducibility of the work that we publish. This form provides structure for consistency and transparency in reporting. For further information on Nature Portfolio policies, see our Editorial Policies and the Editorial Policy Checklist.

Statistics

For all statistical analyses, confirm that the following items are present in the figure legend, table legend, main text, or Methods section.

n/a	Confirmed	
☐	☒	The exact sample size (*n*) for each experimental group/condition, given as a discrete number and unit of measurement
☐	☒	A statement on whether measurements were taken from distinct samples or whether the same sample was measured repeatedly
☐	☒	The statistical test(s) used AND whether they are one- or two-sided<br>*Only common tests should be described solely by name; describe more complex techniques in the Methods section.*
☐	☒	A description of all covariates tested
☐	☒	A description of any assumptions or corrections, such as tests of normality and adjustment for multiple comparisons
☐	☒	A full description of the statistical parameters including central tendency (e.g. means) or other basic estimates (e.g. regression coefficient) AND variation (e.g. standard deviation) or associated estimates of uncertainty (e.g. confidence intervals)
☐	☒	For null hypothesis testing, the test statistic (e.g. *F*, *t*, *r*) with confidence intervals, effect sizes, degrees of freedom and *P* value noted<br>*Give P values as exact values whenever suitable.*
☒	☐	For Bayesian analysis, information on the choice of priors and Markov chain Monte Carlo settings
☒	☐	For hierarchical and complex designs, identification of the appropriate level for tests and full reporting of outcomes
☐	☒	Estimates of effect sizes (e.g. Cohen's *d*, Pearson's *r*), indicating how they were calculated

Our web collection on statistics for biologists contains articles on many of the points above.

Software and code

Policy information about availability of computer code

Data collection	FV10-ASW v.3.0 and Prairie View 5.4: imaging data. Actimetrics FreezeFrame 4: fear conditioning data.
Data analysis	Image J Fiji 1.0: imaging analysis; GraphPad Prism 8 and Microsoft Excel 16.36 : statistical tests.

For manuscripts utilizing custom algorithms or software that are central to the research but not yet described in published literature, software must be made available to editors and reviewers. We strongly encourage code deposition in a community repository (e.g. GitHub). See the Nature Portfolio guidelines for submitting code & software for further information.

Data

Policy information about availability of data

All manuscripts must include a data availability statement. This statement should provide the following information, where applicable:

- Accession codes, unique identifiers, or web links for publicly available datasets
- A description of any restrictions on data availability
- For clinical datasets or third party data, please ensure that the statement adheres to our policy

We declare that all data supporting the findings of this study are provided within the paper and its supplementary information. Underlying data of all figures are provided in the Source Data file with this paper and data are fully available from the corresponding author on request. A source data file is provided with this paper.

Human research participants

Policy information about studies involving human research participants and Sex and Gender in Research.

Reporting on sex and gender	n/a
Population characteristics	n/a
Recruitment	n/a
Ethics oversight	n/a

Note that full information on the approval of the study protocol must also be provided in the manuscript.

Field-specific reporting

Please select the one below that is the best fit for your research. If you are not sure, read the appropriate sections before making your selection.

☒ Life sciences ☐ Behavioural & social sciences ☐ Ecological, evolutionary & environmental sciences

For a reference copy of the document with all sections, see nature.com/documents/nr-reporting-summary-flat.pdf

Life sciences study design

All studies must disclose on these points even when the disclosure is negative.

Sample size	We did not perform power analysis to predetermine sample sizes. Our sample sizes are similar to those generally employed in the field. The sample size is the number of animals, neurons, or dendritic spines examined.
Data exclusions	No samples or animals that were successfully imaged or measured were excluded from the analysis. No data was excluded from the analysis.
Replication	Experiments on fear conditioning and extinction-induced freezing response and fear extinction generalization was repeated at least two times with similar results. Experiments on fear conditioning and extinction-induced spine remodeling and neuronal changes was repeated at least three times with similar experiments.
Randomization	Mice used in the study were randomly assigned to each group to maintain total randomization. Mice in each group were randomly assigned to receive behavioral training and surgery.
Blinding	Acquisition of behavioral and imaging were completely automated and without experimenter involvement. Investigators were blinded to groups allocation during data analysis

Reporting for specific materials, systems and methods

We require information from authors about some types of materials, experimental systems and methods used in many studies. Here, indicate whether each material, system or method listed is relevant to your study. If you are not sure if a list item applies to your research, read the appropriate section before selecting a response.

Materials & experimental systems

n/a	Involved in the study	
☐	☒	Antibodies
☒	☐	Eukaryotic cell lines
☒	☐	Palaeontology and archaeology
☐	☒	Animals and other organisms
☒	☐	Clinical data
☒	☐	Dual use research of concern

Methods

n/a	Involved in the study	
☒	☐	ChIP-seq
☒	☐	Flow cytometry
☒	☐	MRI-based neuroimaging

Antibodies

Antibodies used	Rabbit Polyclonal Anti-RPF (600-401-379), Rockland Immunochemicals. Alexa Fluor 555 Goat anti-rabbit IgG Secondary Antibody (A27039), Life Technologies.

Validation	The antibodies were chosen based on the validation information on the manufacturer's websites.<br>Polyclonal anti-RFP is designed to detect RFP and its variants. This antibody has been tested by ELISA, western blot, IF, and IHC, and is suitable for use in IP, ICC, dual RNA-FISH, iDISCO+, IEM, and FLOW. No reaction was observed against Human, Mouse or Rat serum proteins.<br>Alexa Fluor 555 Goat anti-rabbit IgG Secondary Antibody is validated for Immunocytochemistry, Immunofluorescence. The sensitivity and specificity of each lot is confirmed using ELISA. Minimal cross-reactivity with mouse, rat, human, bovine, guinea pig and donkey IgG is observed.<br>Information about validation can be found at the following links:<br>https://www.rockland.com/categories/primary-antibodies/rfp-antibody-pre-adsorbed-600-401-379/<br>https://www.fishersci.com/shop/products/igg-h-l-goat-anti-rabbit-alexa-fluor-555-secondary-antibody-invitrogen/A27039

Animals and other research organisms

Policy information about studies involving animals; ARRIVE guidelines recommended for reporting animal research, and Sex and Gender in Research

Laboratory animals	Mice at age of 28-30 days were used in behavioral and imaging experiments. Mouse, Thy1-YFP-H line, male and female. Mouse, C57BL/6J, male and female. Mouse, SSTtm2.1(cre)Zjh/J, male and female. Mouse, Thy1.2-GCaMP6S line3, male and female. We provide information on housing conditions for the mice, describing dark/light cycle, ambient temperature and humidity in the manuscript.
Wild animals	This study did not involve wild animals.
Reporting on sex	We did not have a bias toward the gender of animals in all experiments. Male and female mice were both used in this study, similar to the way generally employed in the field.
Field-collected samples	This study did not involve samples collected from the field.
Ethics oversight	Study protocols have been approved by the Institutional Animal Care and Use Committee (IACUC) of New York University School of Medicine and Peking University Shenzhen Graduate School. All experiments were performed in accordance by IACUC at each university.

Note that full information on the approval of the study protocol must also be provided in the manuscript.
